# All-in-one Biocomputing Nanoagents with Multilayered Transformable Architecture based on DNA Interfaces

**DOI:** 10.7150/thno.113059

**Published:** 2025-07-25

**Authors:** Vladimir R. Cherkasov, Elizaveta N. Mochalova, Andrey V. Babenyshev, Maxim P. Nikitin

**Affiliations:** 1 Moscow Center for Advanced Studies, 20 Kulakova St, 123592, Moscow, Russia.; 2 Sirius University of Science and Technology, 1 Olimpiyskiy Ave, 354340, Sirius, Krasnodar region, Russia.

**Keywords:** biocomputing, smart materials, DNA interfaces, HER2/neu cell targeting, strand displacement

## Abstract

The pathogen diversity to infiltrate the host organism highlights the demand for equally sophisticated mechanisms for their prevention. The development of "intelligent" agents with molecular logic capabilities are of great hope, but their full theranostic potential has yet to be realized.

**Methods:** The original concept of nanoagents based on “Biocomputing based on particle disassembly” technology has been extended to nucleic acids (NAs) interfaces and inputs. By exploiting the unique properties of NAs, we designed nanostructures that can implement all basic single- and dual-input logic gates on a unified nanoparticle platform through DNA strand displacement triggered by oligonucleotide inputs. Performance of nanostructures was investigated across various output signal detection formats including specific interaction with nanosized objects and targeting cells.

**Results:** Here, we demonstrate autonomous theranostic biocomputing agents based on nanoparticles and DNA interfaces ("DNA-transformers") capable of executing a functionally complete set of Boolean logic gates (YES, NOT, AND, and OR) within a single all-in-one particle structure. Each DNA-transformer is constructed through a multi-layered self-assembly of nanoparticles via DNA-interfaces. The route of the agent's disassembly induced by the particular combination of the specific ssDNA inputs determines the agents' ability to produce the programmed outputs compatible with theranostic applications such as specific targeting of HER2/neu-positive cancer cells.

**Conclusions:** The developed all-in-one DNA-based nanoagents represent a significant advancement in molecular logic devices, establishing a versatile platform for smart nanoagents equally suitable for diagnostic and therapeutic applications.

## Introduction

The discovery of ever new facts regarding the remarkable diversity of pathogens, and their persistent ability to enhance methods of infiltrating the host organism while circumventing or repressing the immune system, highlights the pressing demand for the creation of equally sophisticated and intricate mechanisms for the prevention and management of infectious and oncological diseases. There is great hope associated with the development of molecular logic devices - "intelligent" agents capable of autonomously performing biocomputing, i.e., conducting a comprehensive analysis of multiple input factors of their microenvironment according to the principles of Boolean logic and launching a programmed, condition-driven, therapeutic response 1-3.

Nucleic acid (NA)-based logic device systems represent a particularly compelling platform. The combination of straightforward Watson-Crick base pairing specificity, well-predicted strand-displacement kinetics, along with inherent biocompatibility and biodegradability renders NA nearly ideal for engineering molecular logic circuits of virtually unlimited complexity 4. This is achieved through the combination of diverse computing units, including molecular switches 5,6, logic gates 7-9, amplifiers 10, neuron-like structures 11,12, etc.

Most of the research in this field is focused on the development of logic molecular structures that have indicator (sensing or imaging) functions, which can detect the presence of specific targets they recognize 13-16. For example, to image the distribution of molecules and chemicals in living cells 17,18 or develop molecular diagnostics in both cellular and cell-free settings 19-23 are examples of such applications, to name a few.

A more structurally complex and much less numerous group consists of agents with therapeutic functionality, i.e. the ability to carry out a controlled impact on the environment, individual cells and entire organs as a result of a logic analysis of one's microenvironment 24-26 via, for example, correction of endogenous gene circuits 27,28 or responsive drug delivery 29-31.

Unfortunately, one of the limiting factors on the way to the real clinical application of such molecular logic devices is the vulnerability of NA molecules to the effects of physiological factors, primarily to degradation by nucleases, which are widely present in the blood and organs of most animals and humans 32,33. To address this issue, one potential solution is to increase the spatial density of NAs, which can create steric hindrances impeding the efficient enzyme activity, without significantly compromising NA functional properties 34-36.

A significant portion of research in molecular computing focuses on the use of DNA-nanostructures, also known as DNA-origami, which are self-assembling precisely structured three-dimensional objects composed of DNA molecules 37-39. Indeed, the literature describes a vast array of nanostructures with various shapes and structures that can carry a useful therapeutic load to physiological targets 40-43.

Some of these structures can perform stimuli-controlled logic calculations of varying complexity by binding to specific cellular receptors in vitro 29,44,45, and even under specialized in vivo conditions 46-48. However, despite significant efforts to enhance the stability of DNA-origami to physiological environmental factors (degrading enzymes, suboptimal ionic composition and ionic strength, etc.) by optimizing the shape 49,50, composition 51-53, delivery formulations 54-57, etc., this problem has not yet been solved, and the safe and effective application of DNA origami under real physiological conditions needs to be carefully tested 26,58,59.

Another promising approach to address the issue of NA-based therapeutic logic structures' stability is their immobilization on nanoparticles 60,61. This technique not only enhances the stability of the NA ligands but also expands the functionality of the resulting agents by utilizing additional unique properties of nanoparticles, such as multimodality, multifunctionality, high therapeutic capacity, and controlled release of cargo. For example, a number of studies have shown the possibility of creating therapeutic NA-based nanodevices for stimuli-controlled cell targeting using metal-organic framework structures 62,63, mesoporous silicon 64,65, magnetic 66,67, metal 68,69 and other nanoparticles.

However, the full theranostic potential of this approach has yet to be realized. The literature does not contain a single example of the implementation of a non-origami nanoparticle-based NA-controlled logic systems that achieve specific cell targeting as a result of performing a complete set of basic (YES, NOT, AND, and OR) logic functions. Moreover, the methods for engineering complex two- or more input gates described in the literature are based on the combination of simple single-input gates implemented on different particles. However, this approach is difficult to implement in vivo due to significant spatial separation of the components, their inhomogeneous elimination by the immune system, and the complex hydrodynamic conditions of the well-developed capillary network 1.

Here we show the feasibility of autonomous DNA-based biocomputing nanostructures (“DNA-transformers”) that are capable of implementing a functionally complete set of Boolean logic gates (YES, NOT, AND, and OR) in a single all-in-one agent and demonstrate their application to DNA-controlled binding to biological targets. Our concept involves the engineering of a self-assembling DNA-assisted shielding layer composed of nanoparticles around a core carrier that has immobilized output ligands. We have demonstrated that the DNA interface's specific structure and composition can be selectively transformed by a particular combination of inputs (oligonucleotides of a specific sequence) through strand displacement in accordance with the inherent logic, leading to a programmed output action that includes highly specific targeting of the model HER2/neu receptors on the surface of cancer cells.

## Results

### Basic concept

In this work, we expand our original concept of constructing "intelligent" ligand-sensitive materials based on controlled disassembly of multilayered protein-mediated nanoparticle structures 2 by implementing interfaces based on nucleic acids (NAs), as another important class of biomolecules.

The original technology ("Biocomputing Based on Particle Disassembly") distinguishes itself through its exceptional adaptability to diverse biochemical compositions of the constituent agents. To our knowledge, it is currently the only molecular (cell-free) biocomputing framework capable of implementing a functionally complete Boolean logic system without intrinsic reliance on DNA-based mechanisms. In this paradigm, computational programs are encoded within the spatial architecture of self-assembled nanoparticles and biomolecules, rather than being contingent upon specific biochemical properties of interfacial interactions. Consequently, the proposed framework is not constrained to a singular input-processing interface and is compatible with a broad spectrum of molecular recognition systems, including antibody-antigen interactions and covalent bonding. Experimental validation has confirmed the functionality of biocomputing agents utilizing diverse recognition interfaces such as antibody-antigen, lectin-glycoprotein, and streptavidin-(imino)biotin systems - capable of processing inputs ranging from ions and small molecules to proteins 2,70.

To overcome limitations of protein-based interfaces and demonstrate a non-protein implementation of this platform, we developed “DNA-transformers” (DTs) - nanoparticle-based biocomputing structures with nucleic acid interfaces. These architectures provide the following key advantages:

- Significantly mitigate the gate leakage effect - an unintended output signal gain in the absence of correct input signal - one of the major factors compromising the reliability of logic systems 71. The all-in-one concept - which ensures assembly of DNA interface components in an optimal stoichiometric ratio - addresses a key cause of leakage in protein-based gates: the inherent difficulty of maintaining precise stoichiometry across all elements of complex protein interfaces, where each component exhibits distinct specificity and collectively defines the resulting logic function.
- Dramatically simplify the design of complex multi-input gates through the use of much more compact, easily reproducible and predictable NA structures. The relatively compact size of nucleic acid molecules enables easy design of multi-input logic structures without significant spatial constraints, simply by adding ligand-sensitive fragments to a single molecule.
- Fine-tuning the gate operation, i.e. balancing the receptor affinity, achieved by regulating the affinity through programmable complementarity of NA-interfaces during synthesis. The precise, base-by-base adjustability of affinity and predictable interaction outcomes between DNA elements (molecular interfaces) makes NA uniquely suited for interface engineering. Protein interfaces lack this capability entirely due to: (a) challenges in primary structure synthesis, (b) proper folding requirements (secondary/tertiary structure formation), making targeted modifications extremely costly, time-consuming, and unpredictable.
- Adoption of a unique linear circuit architecture, greatly facilitating the incorporation of multiple active centers within a single molecule, thereby enabling the realization of larger fan-in for multi-input gates.
- Broadening the spectrum of input signals to encompass the ligands of virtually any nature, including DNA and RNA tumor and viral markers, haptens, etc.

To construct DTs, we incorporate logic gating functionality into self-assembled particle/receptor NA-based interfaces and operation is achieved through input-induced disassembly of the structures on the particle through the toehold mediated strand displacement (TMSD) mechanism.

**Figure 1:**
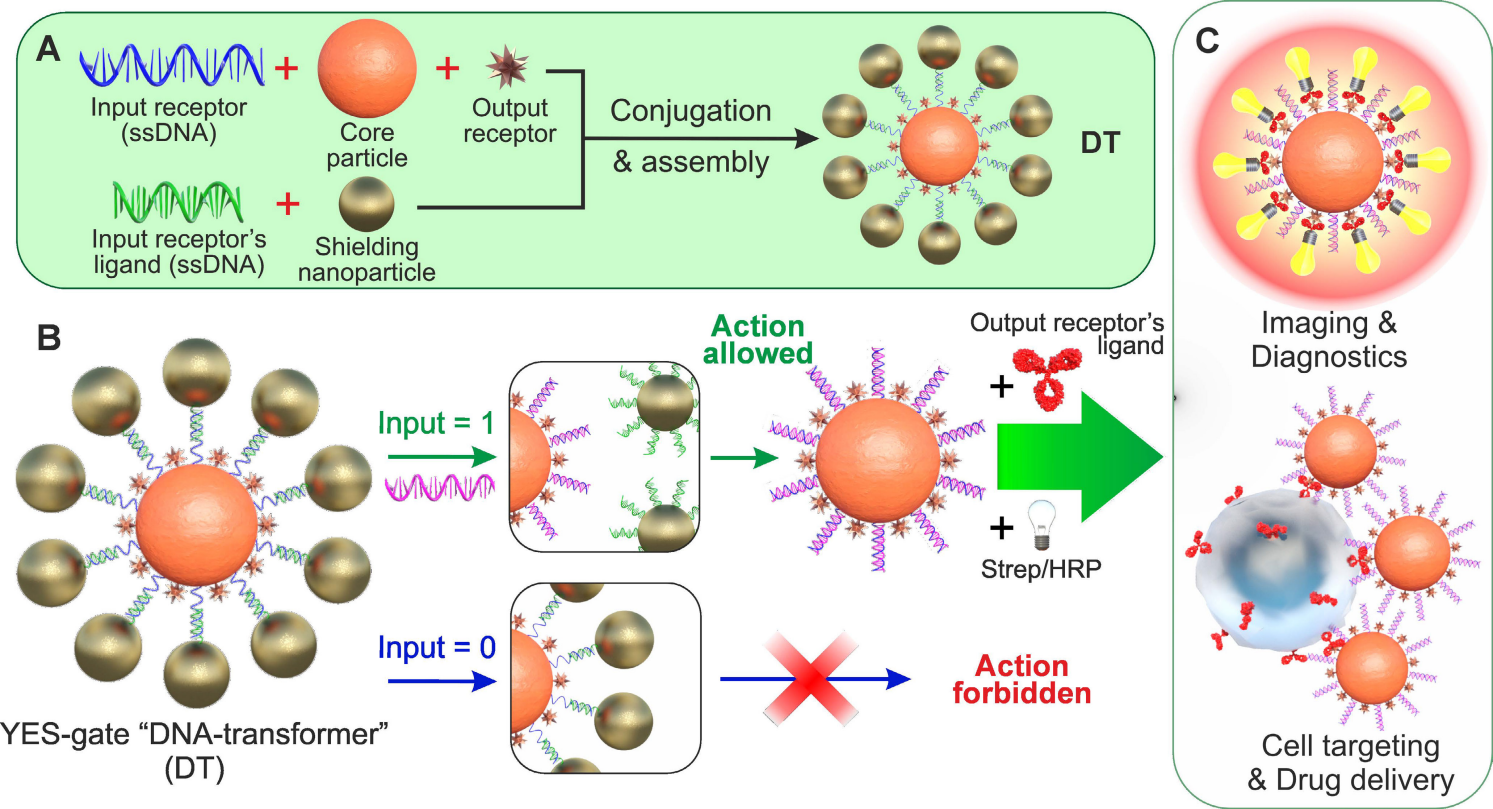
General scheme for implementing the simplest logic YES gate based on DTs. (A) Self-assembly of DTs to implement the YES gate; (B) DT affinity switching scheme by free NA fragments (INPUT); (C) options for generating the output signal as a result of executing the YES function. Strep/HRP denotes a conjugate of streptavidin and horseradish peroxidase.

For example, **Figure 1A** shows a scheme for implementing an NA-based YES gate, according to which only the appearance of a specific (in TMSD terminology - invading strand) input oligonucleotide (INPUT = 1) in the DT environment causes the agent to bind to its target (OUTPUT = 1) replacing partly complementary (protective) strand from NA-duplex. As can be seen from the figure, such a structure consists of particles of two types - central (hereinafter - Core Particle, CP) and shielding nanoparticles (SN), and its formation is the process of self-assembly of these nanoparticles due to the specific interaction of the immobilized specific receptor/ligand pair (i.e., complementary oligonucleotides). In the assembled (initial) state, output oligonucleotide receptors on the surface of the nanostructure (responsible for the formation of the output signal, for example, binding to cells or initiating an indicator reaction) are sterically shielded from interaction with their ligand **(Figure 1B)**. When a specific input oligonucleotide ligand (hereinafter - INPUT) appears, it destroys the connection between the input receptors, the nanocomplex is disassembled, the output receptor becomes available for interaction with its ligand, and the output signal is formed (**Figure 1C**).

**Figure 2:**
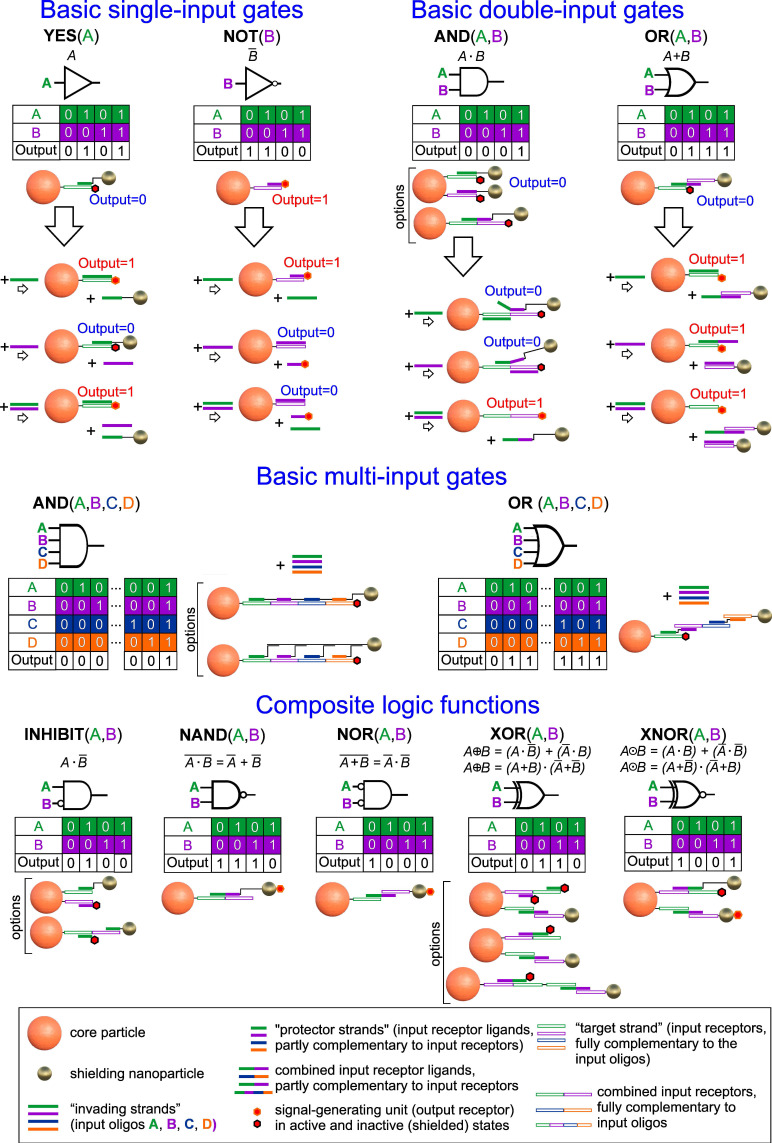
Possible schemes for implementing one-, two- and four-input logic functions using toehold-mediated strand displacement on the DT surface. The detailed scheme of the composite two-input logic gate operating is depicted in **Figure S1**.

The structure of another basic gate, the NOT gate, is ever simpler (**Figure 2**): the core particle carries only an input NA receptor to allow assembly with the complementary strand (input receptor's ligand) conjugated with the output receptor. As result of an input interaction, the output receptor displaced from the core particle. Accordingly, the core particle can bind the output receptor's ligand only if no input is present.

As we showed earlier 2, it is possible to achieve the execution of complex logic functions by combining structures that autonomously implement the basic logic gates via protein interfaces. For instance, two-input AND functions can be implemented by combining YES/NOT gates on a single particle (e.g., [YES(A)]AND[YES(B)] or [YES(A)]AND[NOT(B)]), while OR functions require the combination of YES/NOT/AND-type protein-based structures, each contributing to the net output action. This scheme for implementing logic functions, although simple and employing a minimum set of logic elements, requires a strict balance between the components of composite structures. Specifically, the implementation of logic OR functions necessitates interaction of the individual particles with the target in a precisely defined proportion, which can be challenging to achieve in heterogeneous and dynamically changing environments, such as physiological conditions.

The proposed approach for implementing logic structures based on NA-interfaces is practically free of these limitations (**Figure 2**). By employing the methods described above to shield output receptors through self-organizing NA-interfaces and controlled toehold-mediated disassembly of the shielding layer by a certain combination of input oligonucleotides, it is possible to implement any gate not just on a single particle but even on a single rationally designed NA-sequence. For example, the basic one- (YES/NOT), two- and four-inputs (OR/AND) gates can be combined according to the laws of Boolean algebra 72 to construct virtually any complex function. Some implementation and operational schemes of these functions are presented in **Figure 2** and **Figure S1**, respectively. It is worth noting that almost all of the schemes can be implemented by co-immobilization of several separate molecular constructs, as well as in a unique way by combining them on a single strand immobilized on a CP surface. This approach simplifies assembly and makes the operation of such DTs more predictable and reliable in real conditions.

As depicted in the schemes above, the YES gate, along with other one- and two-input gates (as shown below), relies on the efficient functioning of the DNA interface between CP and SN. Specifically, achieving effective shielding of output receptors, their specific disassembly, and output signal generation are crucial tasks as they determine the difference between the two extreme states of the system (ON/OFF or 0/1) and, consequently, the clarity of logic switching. Therefore, in the initial stage, optimization of the YES gate's operation was carried out, which included adjustments of the nanostructure's composition and the conditions for its formation.

### Optimization of the composition and conditions for the formation of nanostructures for a single-input YES gate

#### Corpuscular components of DTs

**Figure 3:**
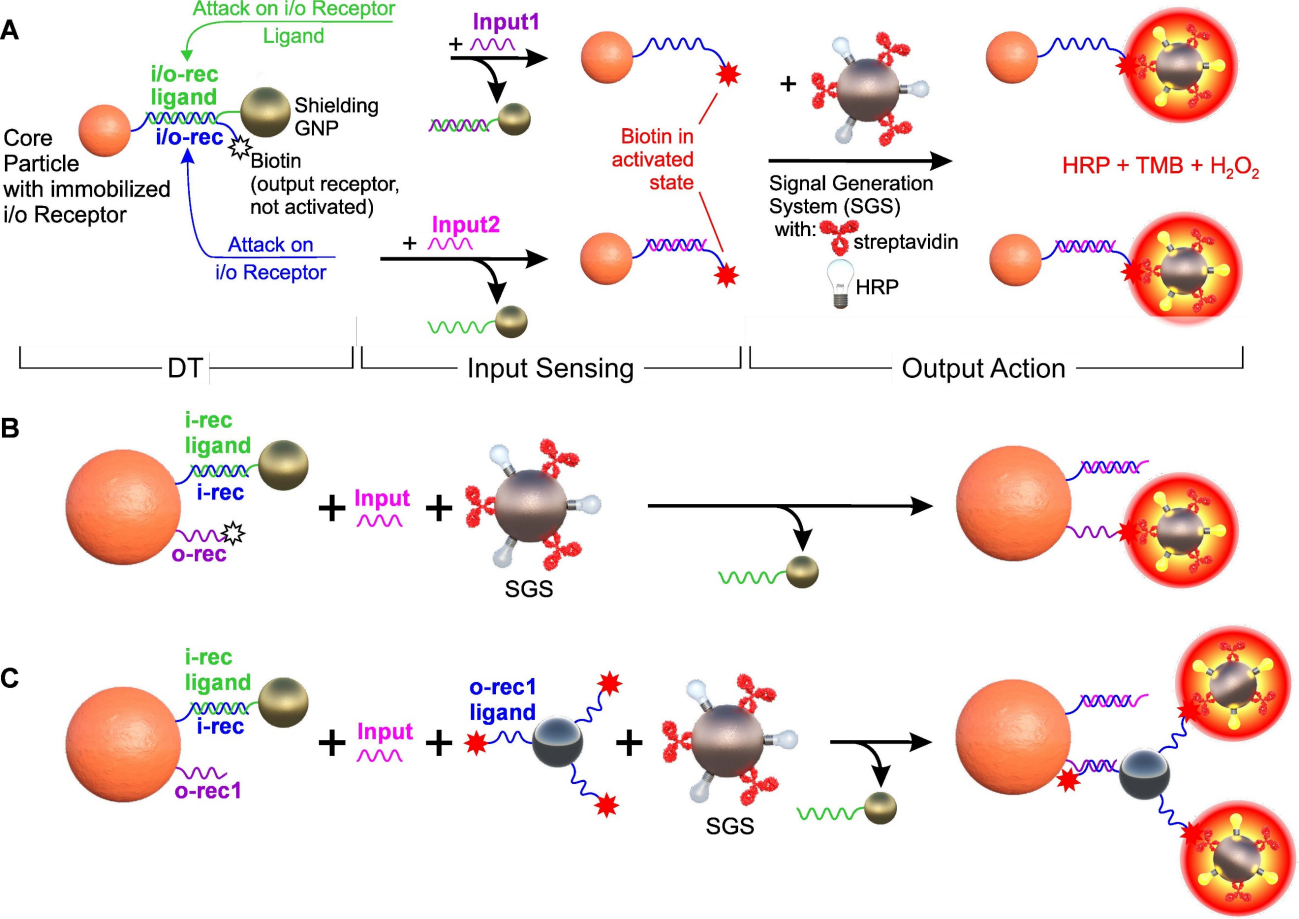
Options for constructing a single-input logic YES gate based on DTs. (A) Input and output receptors, which are certain nucleotide sequences and biotin, respectively, are located on the same DNA molecule (i/o-rec, blue) immobilized on a core magnetic polymer particle (Core Particle). Together with i/o-rec ligand (partially complementary to i/o-rec, green) immobilized on SN, they form “DNA-transformer” (DTs) and biotin in shielded (not active) state. Such a construction can be further disassembled both by attacking with Input1 and/or Input2 (fully complementary oligonucleotides to i/o-rec and to i/o-rec ligand, respectively), forming the Core Particles with non-shielded (activated) biotin. System switching is recorded by a signal generation system (SGS, based on ferrihydrite nanoparticle-streptavidin-HRP conjugate) followed by colorimetric registration of the interaction of HRP with the substrate (TMB/H_2_O_2_). (B) A variant of the separate location of the input and output receptors on two different oligonucleotides, which are co-immobilized on Core Particle. (C) A variant of the separate arrangement of receptors with multiplexing of the output signal, in which the ligand for the output receptor (o-rec1 ligand) is located on the auxiliary SN conjugate. The specific nucleotide sequences used in the scheme are shown in **Figure S2**.

As a starting point, we used an arbitrarily selected sequence (**see Table S1**). To simplify the YES gate design, we positioned both the input receptor (an arbitrary oligonucleotide of specified sequence) and the output receptor (a 3'-end biotin moiety) on the same oligonucleotide (io1), utilizing its 5'-end amino group for covalent conjugation to the carrier particle (CP) (**Figure 3A**). For generating the output signal, we employed a particle-based signal generation system (SGS) that simulates cell targeting through the binding of biotin on CP and streptavidin on SGS, which allows for the binding of DTs to a corpuscular object. The SGS was based on a previously developed optical indicator system 73, which consists of ferrihydrite nanoparticles with surface co-located streptavidin (Strep) and Horse Radish Peroxidase (HRP) moieties. The output signal was determined based on the intensity of staining of the TMB substrate in the presence of hydrogen peroxide due to the reaction with HRP, after thorough magnetic washing to remove any unbound particles of the signal generation system.

Although the proposed approach for implementing DTs is applicable to nearly all types of known nano- and microparticles, in the current studies we utilized particles that facilitate their manipulation and detection. Specifically, we employed polymeric magnetic particles as CPs, which possess necessary functional groups for the covalent immobilization of bioreceptors. These particles can be readily quantified by modern magnetometric methods and have the potential to broaden the functionality of nanostructures, including therapeutic (hyperthermia) and diagnostic (MRI bioimaging) applications 74-76. Gold nanoparticles (GNP) were employed as shielding nanoparticles (SNs) to enable control of the density of the screening layer via plasmon resonance and electron microscopy 77.

The selection of optimal components and their ratio for the formation of DTs was carried out on the example of a YES gate, using both qualitative (SEM data) and quantitative comparison of screening efficiency as a criterion, i.e., ratio of signals in unshielded and shielded form after reaction with the signal generation system.

**Figure 4:**
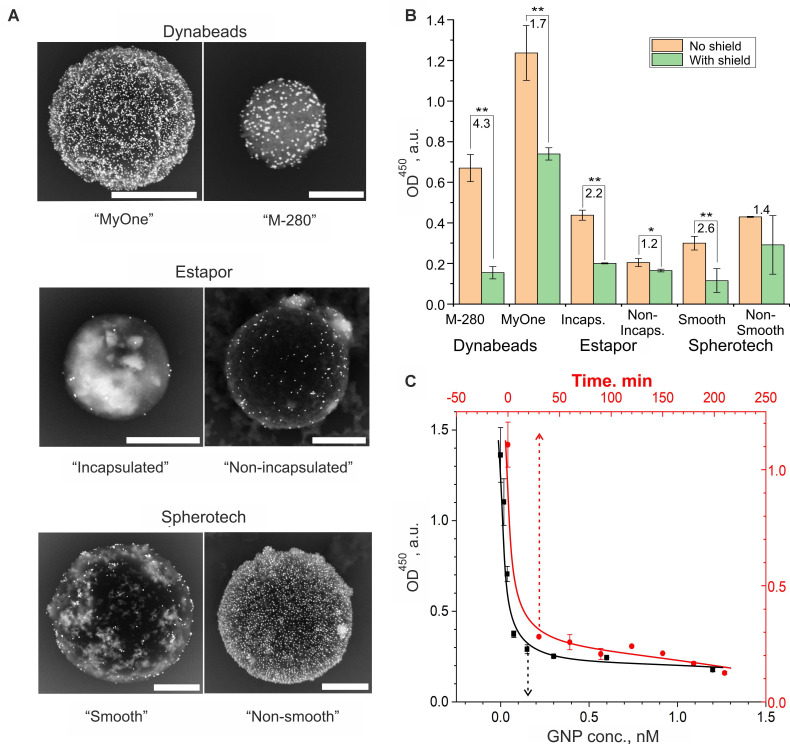
Optimization of the DT assembly conditions for the implementation of the YES gate according to the scheme in **Figure 3A**. (A, B) Comparison of the shielding efficiency of magnetic microspheres of different nature: (A) SEM images (BSE mode, in which heavy metal compounds, primarily gold nanoparticles, are visible). Scale bars correspond to 1 µm; (B) The result of a quantitative comparison of optical signals after interaction with the signal generation system (SGS, conjugate of ferrihydrite particles with streptavidin and horseradish peroxidase) followed by spectrophotometric detection. The numbers indicate the ratio of the signals before (CP) and after (CP+SN) screening of the output ligands on the CPs. One and two asterisks indicate the significance level of the difference (p < 0.05 and 0.01) between shielded and unshielded states of DTs, respectively. The original names of the used commercial particles are used. (C) Dependence of the output optical signal of the reaction of DTs with SGS on the concentration of shielding nanoparticles (black color, incubation time 30 min, CP conc. 3 g/L) and the time of their incubation with CP (red color, GNP and CP conc. 0.15 nM and 3 g/L, respectively). The selected optimal conditions (where the signal reaches a plateau) are indicated by a dotted line.

To optimize the component composition of DTs on a model system (**Figure S3**), we studied the influence of the properties of CPs and SNs, as well as the conditions for the formation of a shielding layer (**Figure 4, S4**). Studies have shown that the screening effect of the output ligands is observed for almost all the studied combinations of CPs and SNs.

The qualitative and quantitative comparison of the impact of CP type reveals that, owing to a synergistic combination of attributes — such as screening efficiency, a well-developed surface yielding high signal-to-noise ratio, consistent colloidal chemical properties, and facile modification potential facilitated by tosylate groups — M-280 microspheres emerge as the most suitable candidate for further investigation. Notably, the precision of the YES gate's functionality is influenced by the concentration of SNs, with a discernible shielding effect observed from 2 nM onwards, following an incubation period of at least 30 minutes (**Figure 4C**). This effect demonstrated minimal dependence on the size of the particles themselves (**Figure S4C**).

Furthermore, the increase of ionic strength within the operational buffer, as anticipated, fostered interactions between interface oligonucleotides and yielded the output ligand blocking, a trend observed at concentrations exceeding 0.2 M (**Figure S4A**). Meanwhile, other buffer properties, encompassing surfactant content and their type, exhibited relatively modest influence (**Figure S4B**).

Thus, for further experiments on the engineering of logic gates, M-280 tosylate microspheres and gold nanoparticles with a diameter of 45 nm were chosen, and DTs was assembled in 1% BSA in PBS at an SN concentration of 0.15 nM for 30 minutes.

#### Output signal generation

As part of the study, three options for implementing the output signal generation system were tested: (a) co-localization of the input and output receptors as a single interface element (**Figure 3A**); (b) placement of the output receptor on a separate spacer immobilized on the CP (**Figure 3B**); and (c) a variant of separate receptor arrangement with an output signal multiplication, in which the output receptor binds to its intermediate ligand placed on an auxiliary particle, followed by binding to signal-developing system (**Figure 3C**).

In the variant of with a separately immobilized output receptor (**Figure 3B**), the input receptor has the previously used sequence with co-localized input/output receptors (i1), but without the terminal biotin. An oligonucleotide of comparable size (30 nt) with an "anchor" amino group at the 5' end for immobilization on CP and a 3'-terminal biotin (o1) was used as the output receptor, which was separately immobilized on CP. The design of this oligonucleotide was optimized to avoid pronounced high-energy secondary structures (< - 2.0 kcal/mol) and absence of complementary sites with the input receptor and its ligand. In the variant of the output signal amplification (**Figure 3C**), complementary sequences were immobilized on the CP and the auxiliary particle, which, upon destruction of the shielding layer, led to their specific binding. In this case, unbound surface oligonucleotides with terminal functional group (e.g., biotin) remain exposed and allow amplification of the signal via signal generation system.

The variety of options for implementing the output signal provides great flexibility in building DTs, depending on their specific areas of application. For example, the simplest design with co-localization of input and output ligands in one oligonucleotide is preferable when using output ligands with small terminal functional group (e.g., biotin), as it simplifies the optimization step and automatically ensures their equimolar availability during DT operation.

In contrast to the first variant, the approach involving separate receptor immobilization - while requiring co-localization of input/output receptors on the same particle and optimization of their ratio (see fluorescence experiments in **Section S1**) - offers greater flexibility in selecting receptor size, nature, and surface conjugation chemistry. Finally, the output signal amplification option, although adding another stage, can significantly increase the output signal and enhance the response sensitivity of the DTs.

The result of the studies demonstrates that with the proper selection of output ligand parameters (including ligand composition, length of its spacer sequence, input/output ligand ratio, etc.), it is possible to achieve reliable operation of the signal generation system for almost all of the options considered in **Figure 3** (for details, see **Section S1**, **Figure S5 and S6**).

#### Input signal sensing

The input signal sensing plays a crucial role in the operation of DTs as it determines the sensitivity and specificity of its operation. Note that, according to the diagram in **Figure 3A**, the disassembly of the DTs can be carried out in at least two ways: through displacement of DNA on the core or on shielding particles. We found that disassembly by displacing of oligonucleotide on the shielding particles is inefficient and poorly reproducible. This is likely due to the formation of a GNP conjugate with the DNA duplex, which has much lower nanoparticle stabilizing ability than single-stranded DNA 78. The potential secondary aggregation of gold particles on the CP surface may have been the cause of distorted results, as observed in previous experiments. Therefore, in subsequent experiments, we mainly used an attack on the input core DNA to disassemble the nanocomplexes.

Based on the previously developed output signal generation system, we conducted a detailed study on the influence of several parameters on the functionality of the simplest YES gate. Specifically, we investigated the following parameters: (i) the composition and length of the input receptors and their ligands (ii), which are responsible for the formation of the CP/SN bond and set the distance between these particles; and (iii) the degree of overlap (i.e., duplex length) of the input receptor and its ligand. We used the NUPACK software (http://www.nupack.org/) 79 to design the corresponding DNA interface components and subsequently checked the resulting sequences for the absence of secondary structures using the Mfold resource (http://www.unafold.org/). The main input parameters for the calculation were (i) the required length of the oligonucleotide chain, (ii) the presence or absence of complementarity with the given sequences, and (iii) the threshold value of the energy of the secondary structure (hairpin) of the generated oligonucleotide. The input receptor design was selected to include "sticky ends" in addition to complementary regions to ensure fast binding kinetics via the toehold-mediated strand displacement mechanism 80.

**Figure 5:**
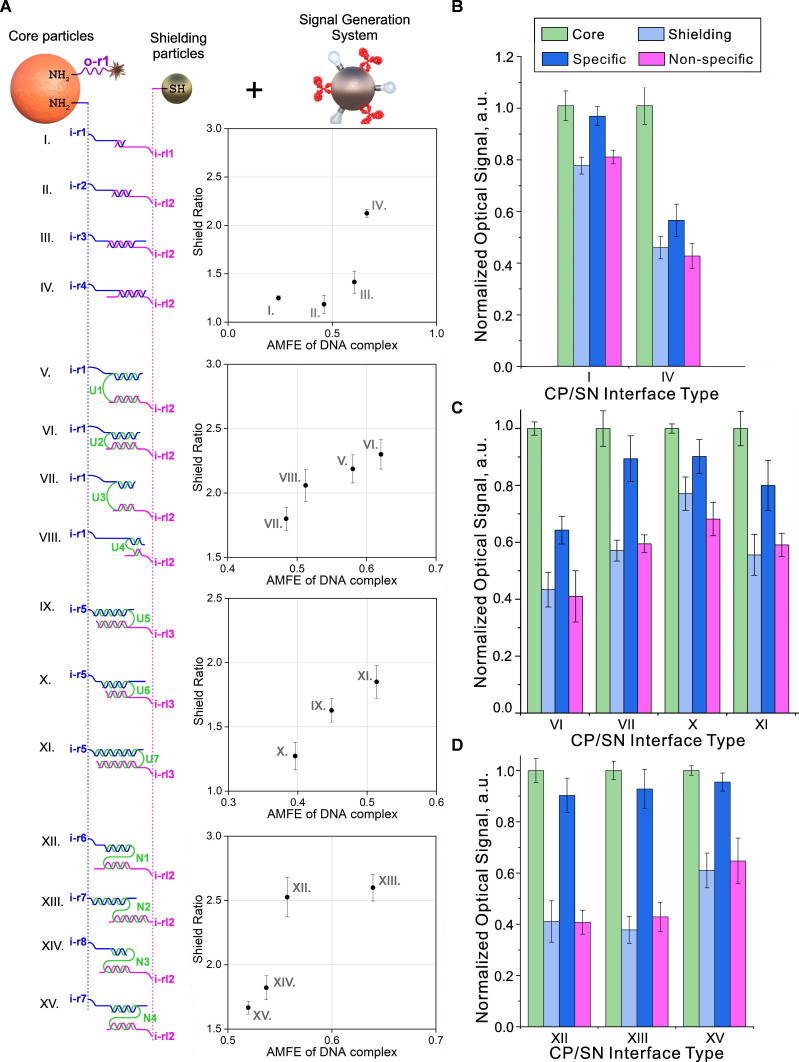
Options for building CP/SN interfaces for implementing a YES gate and their impact on the DT functionality. (A) Tested combinations of randomly generated oligonucleotides immobilized on CP (blue) and SN (pink) and “staples” connecting them (green). The central column shows the Shield Ratio (Ratio of output signal intensity in unshielded and shielded states of DTs) versus Adjusted Minimum Free Energy (AMFE, the ratio of the minimum internal energy of the complex to the total number of nucleotide pairs in the complementary region). The data were obtained using NUPACK software (http://www.nupack.org/), and specific sequences are shown in **Figure S7**. (B-D) The dependence of the optical signal on unshielded (Core) and shielded (Shielding) DTs, as well as in the presence of specific (Specific) and non-specific (Non-specific) oligonucleotides, on the type of DNA interface for several variants of its implementation. The signals were obtained after interaction with the signal generation system and normalized to the value of the signal from the CP in the unshielded state. The sequences used are shown in **Figure S7**.

**Figure 5A and S7** present the sequences and structures that were studied for their possible implementation as the CP/SN interfaces. Depending on the structure of the DNA interface between the central and shielding particles, we divided them into three groups: 1) "direct" shielding (I-IV) and shielding through 2) U- and 3) N-shaped “staples” (V-XI and XII-XV, respectively) as shown in **Figure 5A**. When using "staples", the CPs and SNs are connected through an "intermediate" oligonucleotide (“staple”) with sequences that are partly complementary to the oligonucleotides on both types of particles, while the oligonucleotides themselves do not have complementary regions. The results of the study on the assembly and controlled disassembly processes of the corresponding nanostructures under the same conditions (25°C, 0.5 M NaCl in 2% Tween 20 in PBS) induced by 10^-6^ M INPUT (sequence information can be found in **Figure S7**) are presented in **Figure 5A,B**.

Analysis of the obtained data allowed us to draw the following conclusions regarding the rules for selecting input receptors for gate implementation: (i) the efficiency of YES gates depends on a combination of several parameters for the formation of the CP/SN interface, including the nucleotide sequence, the length of the complementary region, the minimum internal energy of the formed complex, and the total length of the oligonucleotide chain between the central and shielding particles. These parameters interact in a complex way to determine the efficiency of the YES gate. Thus, we found that an increase in the Adjusted Minimum Free Energy (the ratio of the minimum internal energy of the complex to the total number of nucleotide pairs in the complementary region, AMFE) 81 resulted in an increase in the degree of shielding of output receptors (**Figure 5A**). (ii) Surprisingly, we did not find a significant dependence of the efficiency of the subsequent specific disassembly of the complex by fully complementary INPUT from AMFE (**Figure 5B**). (iii) We also found that out of several studied screening layer organization schemes, the highest efficiency of specific disassembly of DTs is provided for "stapled" variants (**Figure 5C**). Despite the high shielding values achieved by the "direct" scheme, the degree of specific disassembly of such nanostructures was low. We explained this observation based on the concentration effect caused by too dense packing of rigid DNA duplexes during the formation of CP/SN interfaces on the CP surface. It is known that this effect can seriously change the kinetics of oligonucleotide binding compared to a homogeneous reaction 82. As a result, "loose" packing on the surface of the particles apparently reduces this effect, leading to higher efficiency of nanocomplex disassembly, especially in the case of N-shaped staples.

Based on the results presented in **Figure 5**, the N-shaped CP/SN interface (**Figure 5D**) achieved the best shielding efficiency. Moreover, this design also provided the most accurate switching between two states (Off/On or 0/1) due to the almost complete disassembly of the nanostructure and restoration of the original signal. The switch, which functioned as a YES gate, was specific and occurred exclusively in the presence of a complementary oligonucleotide.

#### Realization of the main logic gates in a single all-in-one particle

##### Experimental design

In the previous sections, we described individual DTs elements and optimized their operating conditions for implementing a basic logic element with one input - YES gate. To demonstrate the versatility of our approach, we engineered all other logic gates of the functionally complete set - YES, NOT, OR, and AND - which can serve as building blocks for constructing logic functions of virtually any complexity (**Figure 2** and **S1**).

To do this, we designed the corresponding DNA interfaces from scratch, which implemented the indicated logic functions for two fixed, arbitrarily chosen oligonucleotide sequences (InA and InB) with unequal sizes (17 nt and 28 nt, respectively). We designed these sequences using the NUPACK software service while taking into account the following restrictions: (i) lack of complementarity to each other, (ii) complete absence of secondary hairpin structures (ΔG > 0 kcal/mol) (for nucleotide sequences, see **Figure S8-S10**). To construct the signal generation system, we used the variant with signal amplification (**Figure 3C**). Such a system consisted of a pair: (i) the output receptor o-rec1 oligonucleotide, with an amino group at the 5'-end for immobilization on the central particle; and (ii) a partly complementary ligand (o-rec1 ligand), with biotin and a thiol group at the -5', -3' ends, respectively, immobilized on GNP-based auxiliary particle. Like the input receptor/ligand system, the signal generation system oligonucleotides lacked high-energy hairpins and no cross-complementarity with InA and InB INPUTs.

##### YES-gates

**Figure 6:**
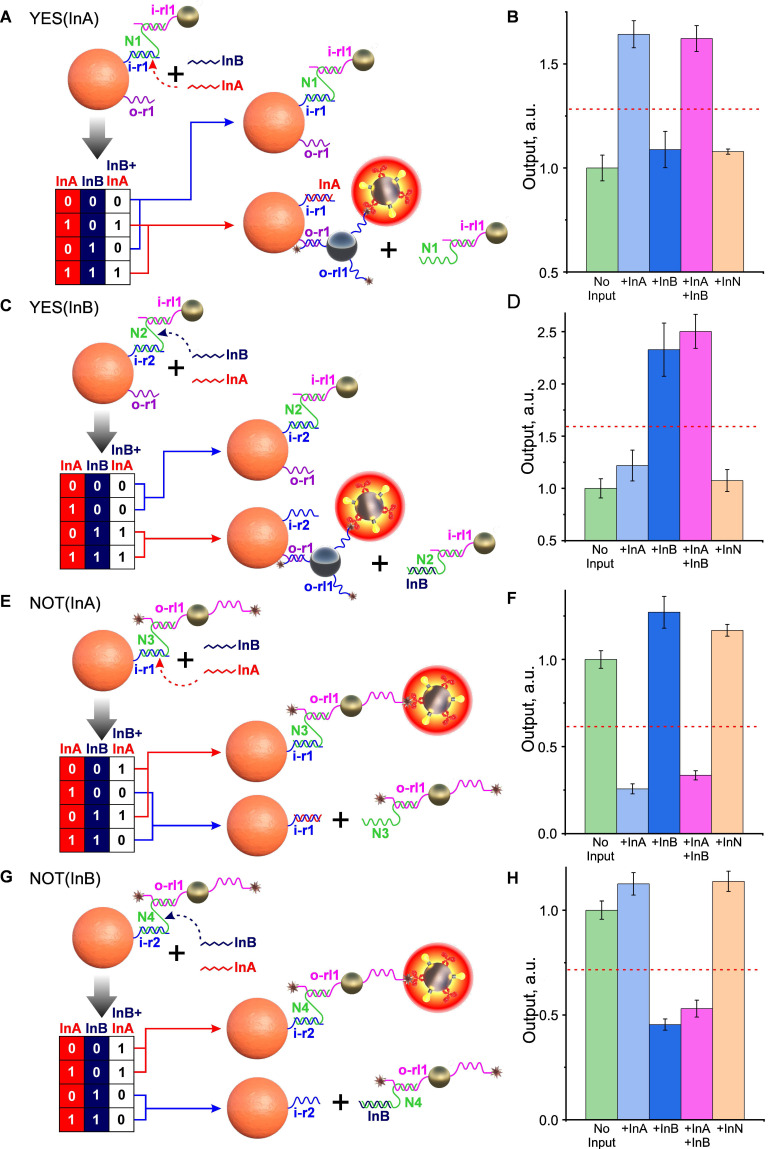
Demonstration of the operation of YES and NOT gates. Implementation scheme for YES (A,C) and NOT (E,G) gates relative to two INPUT DNA sequences (InA and InB). (B), (D), (F), (H) - Dependences of DT optical signals in YES (B,D) and NOT (F,H) gates in response to the presence of specific (InA and InB) and nonspecific (InN) oligonucleotides (10^‑6^ M). The signals are normalized by the corresponding 'No input' signal, and the trigger threshold (geometric average of the maximum and minimum output signals) is indicated by a red dotted line. The sequences of the oligonucleotides used are shown in **Figure S8**.

As a starting point for the design of the above gates, we used our preliminary results on the design of YES gates using an “N-shaped staples” (**Figure 6A**). In this case, the gate construction is based on the selection of the “staple” sequences and on the core particle, which, on the one hand, would ensure the self-assembly of the system and the strong retention of shielding particles to withstand non-specific external factors (including the presence of another INPUT), and on the other hand, enable efficient disassembly by a specific INPUT, thereby initiating binding to the target.

In this case the development of gate design (using the example of a YES gate specific to InA INPUT, **Figure 6A**) followed a sequential process involving the following steps: 1) synthesis of 5'-aminated receptor i-r1 oligonucleotide, fully complementary to InA, and its immobilization on CP; 2) design and synthesis of N1 “staple”, partially complementary to i-r1 and non-complementary to InB; 3) design, synthesis, and immobilization on shielding gold particles of 5'-thiolated oligonucleotide i-rl1, also partially complementary to N1. The design of the N1 “staple” was a critical step in this process, as the extent of its overlap with InA determined the ease of “staple” displacement and, consequently, the functioning of the entire shielding system.

As a result of N1 sequencing optimization, we successfully designed and assembled an DTs that implements a YES (InA) gate, with the nucleotide composition and operational principle depicted in **Figure S8**. Considering the envisioned theranostic applications of our nanoagent and the standard semi-logarithmic dose-response behavior characteristic of many pharmacological compounds (where response is proportional to the logarithm of dose), we defined the threshold as the geometric mean of the gate's maximum and minimum outputs. This threshold value corresponds to the dose of activated core particles that would produce an average therapeutic response 2.

As shown in **Figure 6B**, the signal from the DTs is statistically significant (P < 0.01) only in the presence of InA DNA INPUT (either alone or in combination with non-specific InB), indicating correct operation of the YES gate (YES(InA)). Using a similar approach, we also developed a YES gate that responds to the second input, YES(InB) (**Figure 6C, D**).

##### OR, AND-gates

The unique adaptability of DTs to building interfaces for logic nanoparticle-based structures is especially evident when creating more complex gates, involving the simultaneous participation of two or more input ligands. Indeed, at protein interfaces, complex multi-input gates can only be implemented by immobilizing several receptor/ligand pairs, with each pair responsible for one basic logic element 2. For example, to implement an OR gate, it is important to equalize the contribution of each receptor/ligand pair to the resulting signal. This can create implementation problems for complex protein gates and often requires a compromise between the sensitivity and specificity of such systems.

**Figure 7:**
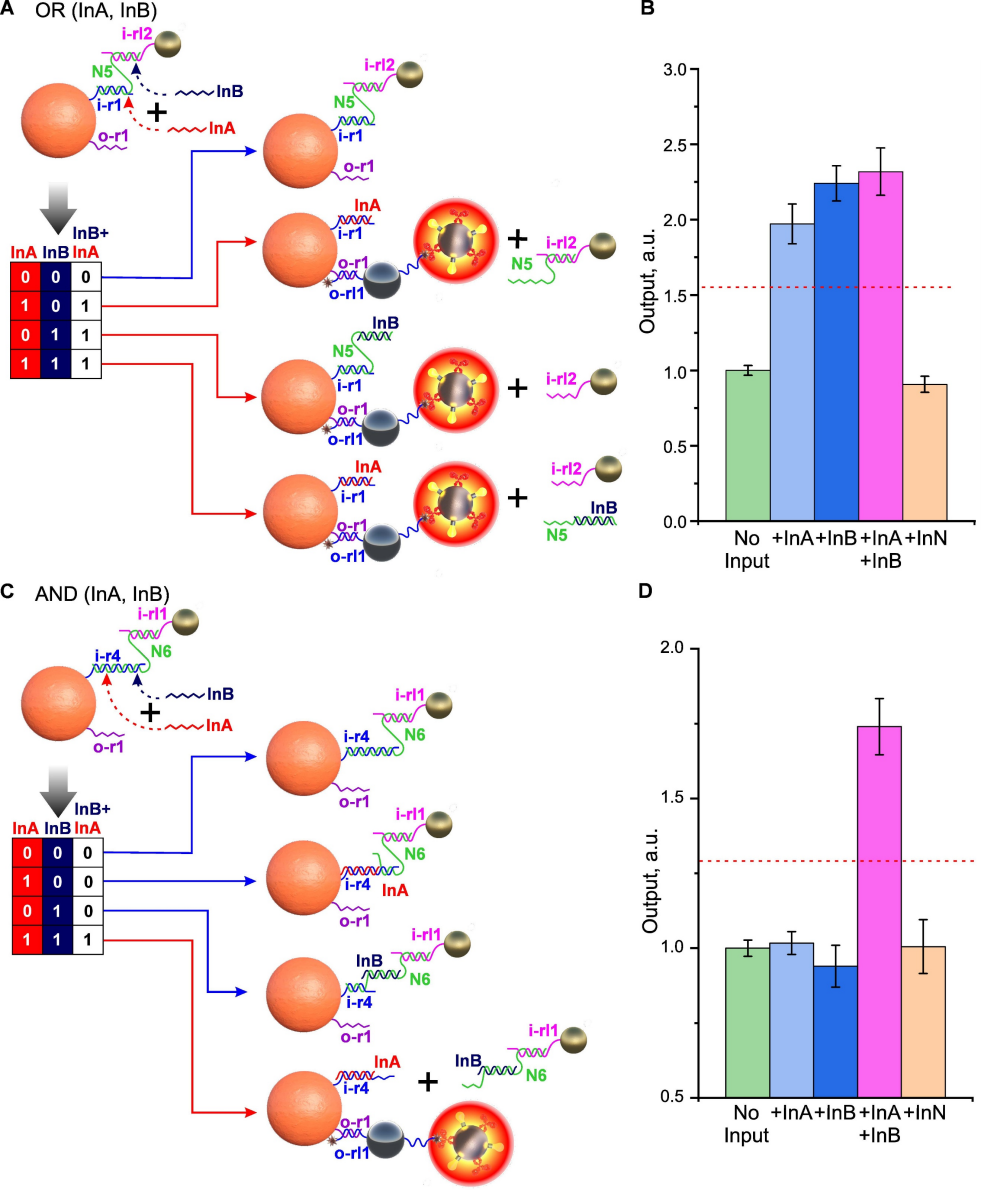
Demonstration of two-input OR and AND gates. Implementation scheme for OR (A) and AND (C) gates relative to the combination of INPUT DNA (InA and InB). Dependences of optical signals of DTs realizing OR (B) and AND (D) gates in response to the presence of specific (InA and InB) and nonspecific (InN) oligonucleotides (10^-6^ M). Absorbance signals are normalized to the corresponding 'No input' signal. The trigger threshold (geometric average of the maximum and minimum output signals) is indicated by a red dotted line. The sequences of the oligonucleotides used are shown in **Figure S9** and **S10**.

In contrast to protein interfaces, complex multi-input gates can be implemented using DNA on a single input receptor/ligand combination. As an example, **Figure 7A** shows one of the possible implementations of the OR gate, in which the removal of the shielding layer occurs by displacing the “staple” N5 from the complex with the input receptor i-r1 and/or displacing the i-rl2 oligonucleotide on SN from N5, thanks to the selected CP/SN interface sequences. The presence of at least one INPUT (InA or InB), as well as their combination, leads to the same output signal comparable in amplitude (**Figure 7B**).

The implementation of an AND gate can be achieved by the cooperative action of two INPUTs, each of which has only partial complementarity to the “staple”/oligonucleotide bond and, therefore, is not able to completely disrupt it (**Figure 7C**). However, with the joint presence and correct selection of the length of non-intersecting complementary fragments, their action adds up and leads to the disassembly of the system. For example, in **Figure 7C**, a variant of AND gate implementation is shown, in which the “staple”/CP bond is disassembled only if both INPUTs are present (**Figure 7D**).

Particularly noteworthy is how DNA interface implementation has significantly improved AND-gate switching precision compared to our previous protein-based system 2. Most remarkably, all false signals (No input, Input A alone, and Input B alone) showed nearly identical values with dramatically reduced variability (CV 4%) - a stark improvement over the protein-based system's performance (CV 23%) where gate leakage was clearly evident.

##### Logic-gated cell targeting

According to a number of recent studies, the presence of various DNA and RNA molecules with different compositions and sizes in physiological media can play a crucial role in regulating body functions. Although the functions of individual members of this family, particularly small RNA and DNA (sRNA/sDNA), are only beginning to be intensively studied 82, there is already evidence of their involvement in regulating transcription processes, chromosome replication, RNA processing and translation, oncogenesis, etc. 84,85. Furthermore, Nikitin highlighted the potentially crucial role of such molecules in regulating cascades of physiological reactions through the recently discovered phenomenon of “strand commutation” 4. Therefore, performing a comprehensive logic analysis of the entire set of such sequences and accurately identifying physiological targets as a result of this analysis is an extremely important task. Solving this task can be equally useful both for fundamental studies of cell interactions, including cancer cells, with the body, and for applied research, such as the diagnosis and treatment of cancer.

To demonstrate the practical potential of the proposed DT construction concept, we applied the developed approach to create stimuli-responsive constructs that selectively target cancer cells based on a logic analysis of their microenvironment for the presence of specific dissolved DNA fragments.

As a model of such targets, we used human epidermal growth factor receptor 2, HER2/neu, which is a clinically significant marker overexpressed by some breast, ovarian, and other cancer cells 86. HER2-positive murine colorectal carcinoma cells CT26 transduced with the HER2 gene 87, human breast adenocarcinoma cells SK-BR-3, and human breast carcinoma cells BT-474 were used, while Chinese hamster ovary cells (CHO) served as a negative control. We achieved specific binding to cancer cells by implementing YES and AND gates using previously developed DNA interface systems (**Figure 6A** and **7C**), where binding to cancer cells occurred for output = 1 and did not occur for output = 0. To demonstrate the flexibility of implementing such logic systems, we used a variant in which all input and output receptors were located on a single oligonucleotide immobilized on CP.

According to the experimental scheme (**Figure S11**), binding to cells was achieved by labeling HER2 receptors with specific antibodies conjugated to streptavidin (anti-HER2 Mab:Str), followed by binding of DT constructs through the streptavidin/biotin bond, which is known as one of the strongest non-covalent bonds in nature 88. In the case of output = 1, the terminal biotin on the output oligonucleotide was deblocked and became available for the formation of a bond with cells; otherwise, biotin remained hidden and no targeting of cancer cells occurred (output = 0).

**Figure 8:**
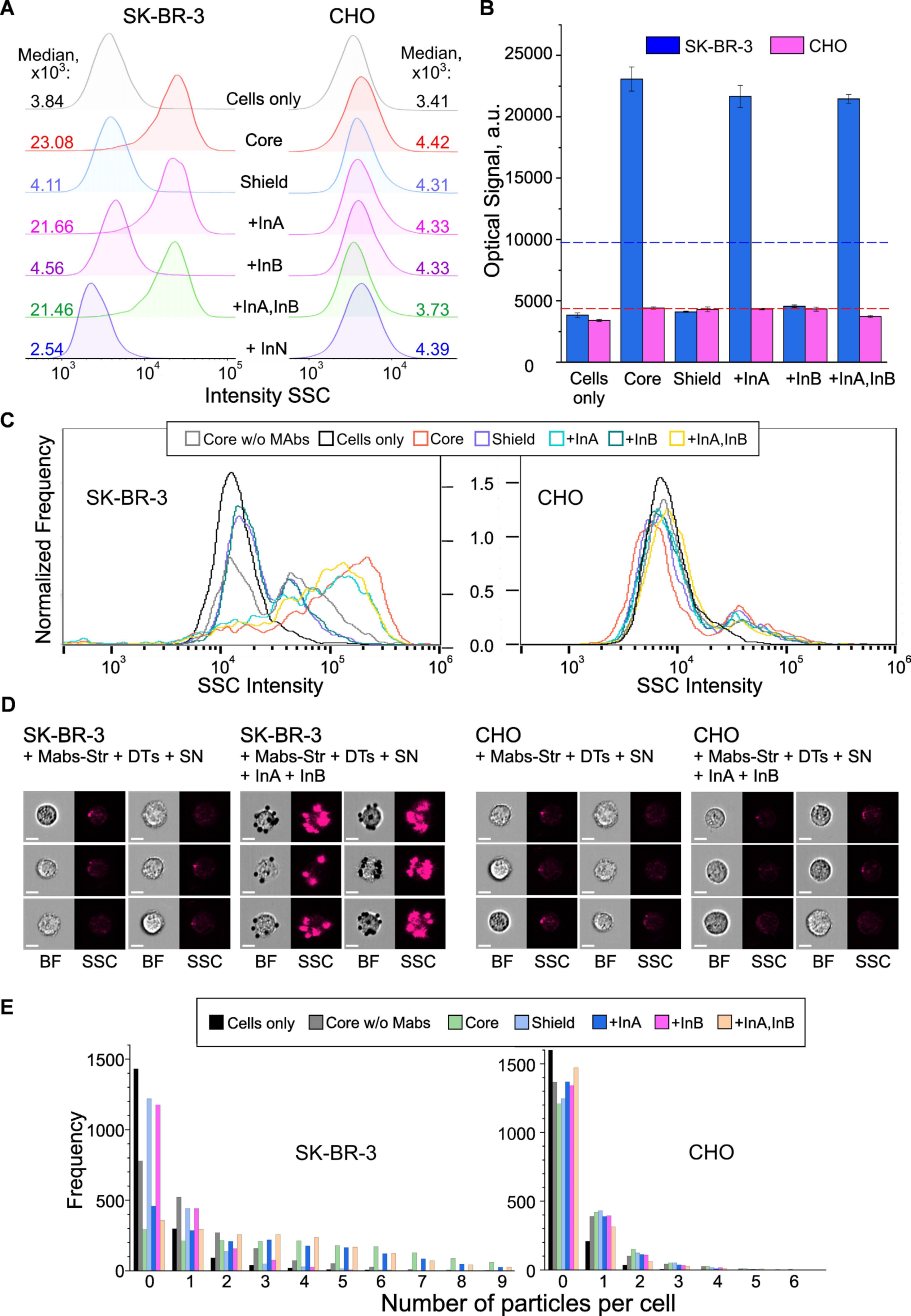
Cell targeting as result of the YES gate implementation. Flow cytometry (A,B) and imaging flow cytometry (C-E) data on registration of the interaction of HER2-positive (SK‑BR‑3) and HER2-negative (CHO) cells with DTs in the presence of specific and non-specific input oligonucleotide. Binding to cells (output = 1) occurred through the interaction of output DT ligands (DNA-bound biotin) with surface HER2 receptors preliminary labeled with anti-HER2/streptavidin conjugate. Histograms of signal distribution in side scatter (SSC) channels (A, C) were generated, and the operation of the YES gate was demonstrated by comparing the medians of the output signals (B). The thresholds for the YES gate (geometric mean of the maximum and minimum output signals) were indicated by a dotted line for both types of the cells. Representative images in bright-field (BF) and SSC channels (D) were recorded, and the specificity of DTs-cell interactions was analyzed using convolutional neural network analysis with the number of bound particles as a quantitative criterion (E). Designations: Cells only - background signal (cells in the absence of particles); Core w/o Mabs - signal from cell interaction with DTs in the absence of preincubation with anti-HER2-Str conjugate (specificity control); Core, Shield - signals from cell interaction with CP and CP+SN components of DTs, respectively; +InA, +InB, +InA,InB, +InN - signals from the interaction of cells with fully assembled DTs in the presence of the first (specific) and second (nonspecific) inputs (10^-6^ M), their joint combination and a nonspecific oligonucleotide of arbitrary composition, respectively. The sequences of the oligonucleotides used are shown in **Figure S9** and **S10**.

**Figure 9:**
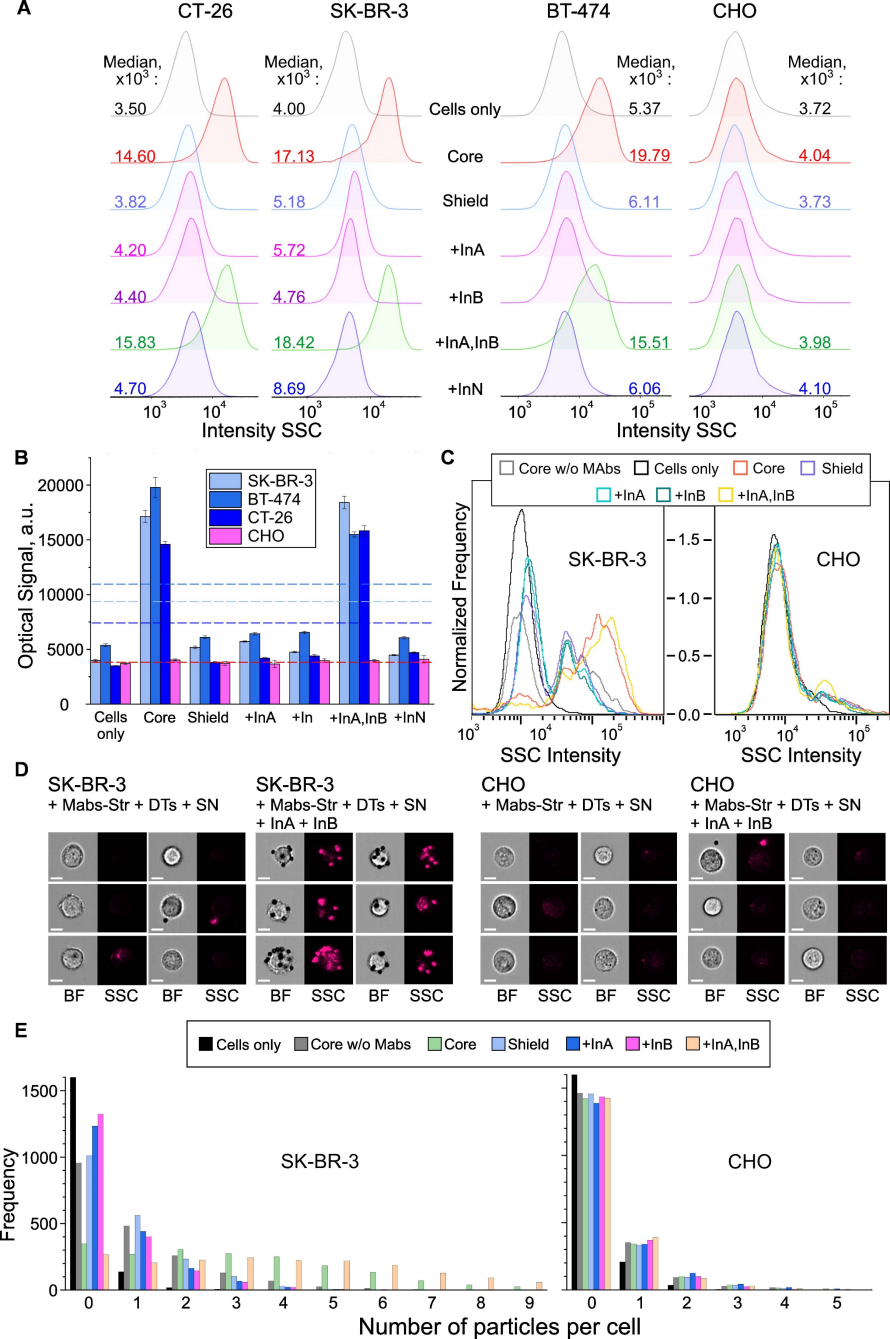
Cell targeting as result of the AND gate implementation. Flow cytometry (A,B) and imaging flow cytometry (C-E) data on registration of the interaction of HER2-positive (SK‑BR‑3, BT-474, CT-26) and HER2-negative (CHO) cells with DTs in the presence of specific and non-specific input oligonucleotide. Histograms of signal distribution in side scatter (SSC) channels (A, C) were generated, and the operation of the AND gate was demonstrated by comparing the medians of the output signals (B). AND-gates switching thresholds are indicated by a dotted line for all types of cells in compliance with color coding; (D) Representative images in BF and SSC channels; (E) Results of convolutional neural network analysis of the specificity of DT-cell interactions. Further details and designations can be found in the caption of **Figure 8**.

To investigate the interaction of cells with DTs, we used cytometric analysis. To increase the reliability and objectivity of the studies, we applied two independent methods: conventional and imaging flow cytometry. According to conventional flow cytometry data (**Figures 8A,B and 9A,B**), particle binding to HER2-negative CHO cells was virtually undetectable both with and without input DNA, as confirmed by identical signal values in side scatter (SSC) channels. In contrast, for HER2-positive cancer cells - SK-BR-3 cells (YES gate) and CT26, SK-BR-3, and BT-474 cells (AND gate) - we observed statistically significant (p << 0.01) SSC signal intensity shifts upon addition of InA (for YES gate, **Figure 8B**) or only the InA+InB combination (for AND gate, **Figure 9B**). Notably, these signal intensities matched those from unshielded DT structures ("Core" in figures), indicating near-complete nanostructure disassembly triggered by chemical inputs. Thus, the DTs demonstrate biological functionality fully consistent with their inherent logic: specific binding only occurs for HER2-positive cancer cells and only in the presence of either one (YES gate) or two (AND gate) specific input DNA molecules.

In addition to recording fluorescence and scattered light, imaging flow cytometry allows collection of up to 120,000 images of individual cells per second 89,90.

Since DT constructs were well detected in the bright-field (BF) and side scatter (SSC) channels of the instruments, no additional fluorescent labels were used. The obtained data not only confirmed the results of conventional flow cytometry (**Figures 8C** and **9C**), but also provided unique information on cell morphology and particle localization on their surface (**Figures 8D** and **9D**). Furthermore, we employed our previously developed convolutional neural network-based image analysis method 90 to quantify particle-cell binding. This approach enables precise discrimination between bound and unbound particles within the field of view (**Figures 8E** and** 9E**), ensuring accurate measurements. Using this technique, we detected ever more distinct differences in binding efficiency depending on the presence of specific input DNA in the samples.

## Discussion

The engineering of ligand-sensitive logic devices with a DNA interface using nano- and microparticles has ample application prospects for cell targeting based on logic-gated analysis of soluble biomolecular inputs. As new knowledge about the physiological role of the soluble onco- and other markers in the body becomes available, the biomedical development horizons of these devices will continue to expand. In this work, we have demonstrated that the combination of particle properties with the unique computational capabilities provided by nucleic acids can create structures with new functionality that is yet unattainable for logic nanodevices with protein interfaces.

In particular, the linear molecular structure and the potential for implementing complex sequences bound together by well-predictable forces present exclusive prerequisites for the development of gates with multiple fan-ins (e.g., AND). Although our primary objective did not involve substantiating this aspect, our findings indicate the potential for the disassembly of single-staple structures by at least four distinct inputs. Moreover, using the well-known ability of NA molecules to form complex structures, for example, by introducing additional staples, one can potentially increase the number of inputs of a smart nanoagent, without significant changes in its output signaling system.

Another unique feature of NAs as an interface for creating DTs is the ease and flexibility in the way complex gates can be designed. For example, the "staple-like” design of DNA interfaces allows for several options for disassembling the shielding layer by breaking the “staple”/oligonucleotide bonds and correspondingly switching the DT activity. This disassembly can be achieved both by displacement of a DNA fragment with a lower affinity by its more complementary analog (OR gate, **Figure 7A**) and/or by the cooperative action of several shorter non-overlapping sequences (AND gate, **Figure 7B**). Since DNA duplex disassembly can be carried out by displacement of both forward and reverse sequences, and the number of “staples” of different composition connecting CP and SNs can be practically unlimited, the number of options for controlling DT activity using several INPUTs or their arbitrary combination can also be very large. Such flexibility in the choice of options for DNA interface implementation provides wide opportunities for regulating the specificity of DTs, the complexity of its response to multiple material stimuli and a wide range of the complex design choices.

On the other hand, the process of creating DTs has its own characteristics that must be taken into account during design. Even under optimal conditions (high ionic strength and room temperature), the bimolecular association rate constants for DNA 91,92 and RNA 93,94 are only 10^-6^ - 10^-7^ M^-1^s^-1^, which indicates the equilibrium nature of such interactions and their strong depending on the hybridization conditions, including the concentration, composition and presence of secondary structures of the reacting components, temperature, ionic strength, pH, etc. 95.

The complexity of electrostatic, conformational, and molecular interaction phenomena that arise in the crowded interfacial environment can affect both thermodynamics and kinetics of DNA assembling/disassembling process on the nanoparticle surface 96,97. Besides, though the thermodynamics of NA hybridization have been well studied, the kinetics of hybridization remain poorly understood, and only a few models or algorithms have been reported that can satisfactorily predict hybridization rate constants 82,95.

Finally, upon immobilization on nanoparticles, NA molecules may undergo distinctive transformations and manifest behaviors not typically associated with free molecules. These include phenomena such as the previously observed by us "masking" of macromolecular fragments 69, enhanced resistance to enzymatic activity 60,61, various sequence- and structure-dependent nanoparticle stabilization efficacy 98-100, among others.

Taken together, these facts significantly reduce the accuracy of predicting the thermodynamic and kinetic parameters of interaction between oligonucleotides using available approaches and, ultimately, complicate the design of DTs, making it more empirical.

In this regard, great hope for simplifying the design of complex DT-based gates can be expected from the recently proposed “strand commutation” approach 4. In an extension of the “classical” Watson-Crick theory of the construction of the DNA molecule and the transfer of information through completely complementary interactions, Nikitin demonstrated the existence of the alternative “molecular commutation” mechanism of information processing with non-complementary NAs and other molecules. We believe that the postulates formulated in this work will significantly propel the advancement of a number of areas in modern molecular biology and bioinformatics, including improving the accuracy of modern methods for designing and predicting the interaction of NA molecules under various, including physiological, conditions.

## Conclusion

We have shown implementation of various logic gates on a relatively simple structures, including a single rationally designed DNA molecule immobilized on a carrier particle. The wide range of possibilities for combining logical molecular elements and the flexibility of the scheme for constructing logical functions of almost unlimited complexity make it possible to create smart nanoagents with potential in vivo applications. Due to the broad analytical potential of nucleic acids, such structures may respond in a complex way to various combinations of external chemical stimuli of various natures, including low molecular weight physiologically active haptens, as well as full-sized biomolecules such as proteins and nucleic acids.

We believe that the use of a simple and flexible approach for constructing molecular logic devices with a wide range of output actions will open a new milestone in the development of "smart", ligand-dependent nanoagents that are equally suitable for diagnostic and theranostic applications.

## Experimental Section

### Materials

Tosylactivated magnetic microspheres were purchased from Invitrogen, USA (Tosylactivated Dynabeads M-280, 2.8 μm and MyOne, 1 µm). Other magnetic carboxyl-activated beads were purchased from Merck Millipore Co, USA (0.3, 1, 2 µm Encapsulated and Non-Encapsulated Estapor Magnetic Microspheres) and Spherotech, Inc., USA (3.4 and 3.7 µm Sphero Magnetic Particles with “Smooth” and “Non-Smooth” surfaces). Horseradish peroxidase was obtained from ThermoFisher Scientific, USA. Streptavidin, bovine serum albumin, phosphate-buffered saline tablets (pH = 7.4), sodium hydroxide, 2-(N-morpholino)-ethanesulfonic acid (MES) hydrate, 4-(2-Hydroxyethyl)piperazine-1-ethanesulfonic acid (HEPES), 2-Amino-2-(hydroxymethyl)-1,3-propanedio (Tris), N-(3-(dimethylamino)propyl)-N-ethylcarbodiimide hydrochloride (EDC), N-hydroxysulfosuccinimide sodium salt, 3,3',5,5'-tetramethylbenzidine, boric acid, polyethylene glycol sorbitan monolaurate (Tween20), sodium phosphate mono- basic dihydrate, sodium phosphate dibasic, dithiothreitol, sodium chloride, 2‑iminothiolane, and 3-maleimidobenzoic acid N-hydroxysuccinimide ester (MBS) were of reagent grade and were purchased from Sigma-Aldrich, Germany. Milli-Q-grade water (Merck Millipore, France) was used in the preparation of aqueous solutions. Chloroauric acid was purchased from Dragtsvetmet, Russia. All thiol-modified oligonucleotides were synthesized by Syntol Ltd. (Moscow, Russia). Humanized monoclonal antibody against the cell-surface receptor HER2/neu Trastuzumab (Herticad) were obtained from Biocad Ltd., Russia. Other amine-, biotin-and fluorophore-modified and unmodified oligonucleotides were purchased from Lumiprobe RUS Ltd. (Moscow, Russia). All reagents were HPLS purified and used without additional purification. Nucleotide sequences of the DNA used in this study are listed in **Tables S1**.

### Methods

#### Particle synthesis

Gold nanoparticles (GNP) and paramagnetic iron oxyhydroxide (ferrihydrite) carboxymethyl dextran (CMD)-coated nanoparticles were synthesized by previously reported methods 69. The particle size and size distribution were monitored using dynamic light scattering (Malvern Zetasizer Nano ZS, Malvern Instruments Ltd., UK) and nanoparticle tracking analysis (AstraTrace, Abisense, Russia), which yielded well-converging results.

#### Conjugation of particles

Core magnetic particles were synthesized by modification of corresponding microspheres with amino-group terminated oligonucleotides following the particle manufacturer's protocol via direct reaction in basic buffer (for tosylactivated magnetic microspheres) or carbodiimide chemistry (for carboxyl-modified magnetic beads, i.e. Estapore and Spherotech particles). The conjugates of ferrihydrite nanoparticles with streptavidin and horseradish peroxidase as a component of Signal Generation System (SGS) were obtained using the carbodiimide method as described elsewhere 70. To produce shield nanoparticles (SN), the conjugates of gold nanoparticles with corresponding thiolated DNA were prepared by the “salt aging” method as reported previously 101.

#### Antibody-streptavidin conjugation

Conjugate of humanized monoclonal antibody against the cell-surface receptor HER2/neu Trastuzumab (Herticad) with streptavidin (anti-HER2 Mab:Str) was prepared by thiolation of streptavidin with Traut's reagent (2-iminothiolane) followed by cross-linking with Trastuzumab using a heterobifunctional MBS (3-maleimidobenzoic acid N‑hydroxysuccinimide ester) linker according to the previously reported protocol 69.

#### Assembly of “DNA-transformers” (DTs)

The assembly of DTs was performed by sequentially adding and incubating the corresponding components of the system at a concentration of 10 μM, in the following order: core particle, “staples” (if needed), and shielding gold nanoparticles. Incubation was carried out at room temperature in 0.5 M NaCl, 2% Tween PBS buffer for 0.5 hours with gentle stirring on a rotator. After each step, unbound reagents were removed by magnetic separation on a magnetic plate, followed by resuspension in PBS by manual shaking.

#### Validation of conjugation efficiency

Assessment of input and output oligonucleotide conjugation efficiency on core particles and confirmation of their co-localization of input/output oligonucleotides on the same particle and determine their ratio was performed by fluorescence microscopy (Axio Observer A1, Carl Zeiss, Germany), and validated using LumoTrace FLUO bioimaging system (Abisense, Russia).

#### Performance of biocomputing structures

The demonstration of ligand-specific disassembly of DTs and the formation of a preprogrammed output signal was carried out in two stages. At the first stage, ligand-dependent disassembly of DTs was carried out at 37°C in PBS with constant stirring on a rotator for 1 hour in the presence of INPUT oligonucleotide (10 μM). Then the reacted DTs was precipitated on a magnet, washed in PBS, and resuspended in BSA (40 μL, 1% in PBS) with the FeH-Str:HRP conjugates (Signal Developing Particle System) and incubated for another 15 min under the same conditions. The solution was magnetically washed in PBS, and then TMB substrate (60 μL) was added to the pellet and incubated for 5 min. The solution was precipitated on a magnet, supernatant (50 μL) was transferred into a 96-well enzyme-linked immunosorbent assay plate, and H_2_SO_4_ (50 μL, 2 M) was added to stop the reaction. The absorption signal at 450 nm was recorded by CLARIOstar multimode microplate reader (BMG Labtech, Germany).

#### Electron microscopy studies

The specimens for scanning electron microscopy (SEM) were obtained by magnetic washing with PBS (five times) and with Milli-Q water (five times) with 20 min incubation between the washings. The final suspensions were transferred onto a silicon wafer pretreated with a “piranha” etching solution (1:3 (v/v) mixture of 25% hydrogen peroxide and 98% sulfuric acid) to remove organic impurities followed by rinsing with acetone (three times) and ultrapure isopropanol (three times). The SEM images were acquired with field emission scanning electron microscope Tescan MAIA3 (Brno, Czech Republic) and ZEISS Crossbeam 550 (Germany).

#### Cell culture

HER2-positive murine colorectal carcinoma cells CT26 87, human breast adenocarcinoma cells SK-BR-3, human breast carcinoma cells BT-474 and Chinese hamster ovary cell line CHO were cultured in DMEM/Ham's F12 (1:1) essential media (PanEco, Russia), containing 100 units/mL penicillin-streptomycin (PanEco) and supplemented with 10% fetal bovine serum (FBS, Hyclone, USA) and 300 mg/L L-glutamine (PanEco).

#### Cytometric assay

For cell targeting experiments, 0.25 million of HER2/neu-positive cells (SK-BR-3, BT-474 or CT26) and HER2/neu-negative CHO as control were incubated in 25 μL 1% BSA in PBS, with anti-HER2/neu-Str (5 μL, 20 μg/mL) for 15 min and were washed in PBS. 5 µL of pre-assembled DTs A (ca. 20 µg) was mixed with oligonucleotides at a concentration of 1 μM in 16 µL 1% BSA and 1 M NaCl in PBS. After 15 min incubation and three washes in PBS, the as-prepared solution (5 μL) was added to the cell suspension and was incubated for 15 min. Then the resulting samples were washed in 1% BSA in PBS.

The cytometric assay was conducted using two independent methods: conventional and imaging flow cytometry. Conventional flow cytometry was conducted with Novocyte 3000 VYB device (ACEA Biosciences, USA) using the side scattering parameter SSC-A for particle detection. At least 20,000 events were collected. Imaging flow cytometry was performed on the Amnis ImageStream X Mark II (Luminex Co., USA) device equipped with 40× objective and 785 nm laser for side scatter measurements (0.5 mW). Focused single cells were gated during acquisition within the Amnis INSPIRE software: (i) by bright-field gradient RMS (40-100); and then (ii) by bright-field area (240-900) vs aspect ratio (0.75-1.0). For each sample, 3000 gated events were collected.

#### Data processing

All experiments were performed at least in triplicates. In all graphs, the values represent the average. The 95% confidence interval for each mean is between the error bars on the plots. The results are presented as a mean standard deviation. Statistical significance between two groups was determined using unequal variances Welch's t-test. P values < 0.05 and < 0.01 were denoted as * and **, respectively.

## Supplementary Material

Supplementary figures and tables.

## Abbreviations

AMFE: total number of nucleotide pairs in the complementary region
ATP: adenosine triphosphate
BF channel: bright-field channel of cytometer
BSE: back-scattered electrons
CHO: Chinese hamster ovary cells
CP: core particle
CV: coefficient of variation
DNA: deoxyribonucleic acid
DTs: DNA-transformers
GNP: gold nanoparticle
HRP: horse radish peroxidase
InA, InB: specific input oligonucleotides
InN: non-specific input oligonucleotide
MRI: magnetic resonance imaging
NA: nucleic acid
PBS: phosphate-buffered saline
RNA: ribonucleic acid
SEM: scanning electron microscope
SGS: signal generation system
SSC channel: side scatter channel of cytometer
SN: shielding nanoparticle
TMB: 3,3′,5,5′-tetramethylbenzidine
TMSD: toehold mediated strand displacement
