# Supplementary material for: All-in-one Biocomputing Nanoagents with Multilayered Transformable Architecture based on DNA Interfaces
Source: Theranostics. 2025 Jul 25;15(16):8451–72. doi: 10.7150/thno.113059 (PMC12374668; doi:10.7150/thno.113059)
Supplement: Supplementary file 1 — Supplementary figures and tables. [file thnov15p8451s1.pdf]

Supplementary material

All-in-one Biocomputing Nanoagents with Multilayered Transformable Architecture based on DNA Interfaces

*Vladimir R. Cherkasov, Elizaveta N. Mochalova, Andrey V. Babenyshev, and Maxim P. Nikitin**

Table S1. Sequences of DNA oligonucleotides used

Figure	Oligonucleotide Designation	Sequence, modifications
3a, S2a	i/o-rec	Bio-ACCTGGGGGAGTATTGCGGAGGGAGGTGA-NH2
	i/o-rec ligand	CTCCCTCCGCAATACTCCCCGTCCATGTA-SH
	Input1	TCACCTCCCTCCGCAATACTCCCCCAGGT
	Input2	TACATGGACGGGGAGTATTGCGGAGGGAG
3b, S2b	i-rec	Bio-ACCTGGGGGAGTATTGCGGAGGGAGGTGA-NH2
	o-rec	Bio-CCAACCCATGATTACAGTGAGCACGACAGA-NH2
	i-rec ligand	CTCCCTCCGCAATACTCCCCGTCCATGTA-SH
	Input	TCACCTCCCTCCGCAATACTCCCCCAGGT
3c, S2c	i-rec	Bio-ACCTGGGGGAGTATTGCGGAGGGAGGTGA-NH2
	o-rec1	TTAGCTAGAATACAGAAGTGCGTTGATA-NH2
	i-rec ligand	CTCCCTCCGCAATACTCCCCGTCCATGTA-SH
	Input	TCACCTCCCTCCGCAATACTCCCCCAGGT
	o-rec1 ligand	SH-AACGCACTTCTGTATTCTACACTTCTGGA-Bio
S3	i/o-rec	Bio-ACCTGGGGGAGTATTGCGGAGGGAGGTGA-NH2
	i/o-rec ligand	CTCCCTCCGCAATACTCCCCGTCCATGTA-SH
S5	i-rec	ACCTGGGGGAGTATTGCGGAGGGAGGTGA-NH2
	o-rec(ROX)	Bio-CCAACCCATGATTACAGTGAGCACGACAGA-NH2
	i-rec ligand	CTCCCTCCGCAATACTCCCCGTCCATGTA-SH
	Non-specific	CATGAGGGGCGGGGACGCCGCCTGTCT-SH
S6	i-r1	Bio-ACCTGGGGGAGTATTGCGGAGGGAGGTGA-NH2
	o-r1	Bio-CCAACCCATGATTACAGTGAGCACGACAGA-NH2
	o-r2	Bio-TCTACATATCACTTC-NH2
	o-r3	Bio-(PEG)10-TCTACATATCACTTC-NH2
	o-rl1	TCTGTCGTGCTCACTGTAATCATGGGTTGGTGT
	o-rl2	GAAGTGATATGTAGA
5, S7	i-r1	CCAACCCATGATTACAGTGAGCACGACAGA-NH2
	i-rl1	ATGGGTTGGTGTGGTTGGGATTACAGTCGTAGGGTATTGAATGTGGA-SH
	i-r2	AGTATTGCGGAGGGAGTTGCGGAGGGAGGTGA-NH2
	i-rl2	CTCCCTCCGCAATACTCCCCGTCCATGTA-SH
	i-r3	ACCTGGGGGAGTATTGCGGAGGGAGGTGA-NH2
	i-r4	TACATGGACGGGGAGTATTG-NH2
	i-r5	GAGGAAGGTCATGATTACAGTCGTAGGGTATTGAATGTGGAAAA-NH2
	i-rl3	ACACCAACCCATGATTACAGTGAGCACGACAGA-SH
	i-r6	NH2-CTTATTCAATACCCTACG

	i-r7	NH2-CTTTCCTACATCTCCACATTCAATACCCTACG
	i-r8	NH2-CTTTCCTACATCTCCAC
	U1	TGGAGGGGGAGTATTGCGGAGTCTATGGATATAAATTCGTGCTCAGTGTAATCATGGGT
	U2	TGGAGGGGGAGTATTGCGGAGATATAAATTCGTGCTCAGTGTAATCATGGGT
	U3	TGGACGGGGAGTATAATGTCACTAAACTACACTGTAATCATGGGT
	U4	TGGACGGGGAGTATCTAAAGTACACTGTAATCATGGGT
	U5	CCACATTCAATACCCTACGAATTTACTCTAATCATGGGTTGGTGT
	U6	ATTCAATACCCTACGAATTTACTCTAATCATGGGTTG
	U7	TTTTCCACATTCAATACCCTACGACTGTAAATTTACTCTAATCATGGGTTGGTGTGGGTTGGTGT
	N1	TGGACGGGGAGTATTGTAAAGTAGTAGGTCGTAGGGTATTGAAT
	N2	TGGACGGGGAGTATTGCGGAGGTAAAGTATATTGAATGTGGAGATGTAGG
	N3	GGGGAGTATTGCGGAGTTGTGGATATTTAGTGGAGATGTAG
	N4	GGGAGTATTGCGGAGGTAAAGTAGTAGGTATATTGAATGTGGAGA
6, 7, S8-S10	i-r1	NH2-CTTTCCTACATCTCCAC
	i-r2	NH2-CTTATTCAATACCCTACG
	i-r3	CGACCTACTACTTTATGTC-SH
	i-r4	NH2-CTTTCCTACATCTCCACATTCAATACCCTACG
	o-r1	TTAGCTAGAATACAGAAGTGCGTTGATA - NH2
	o-rl1	Bio-AACGCACTTCTGTATTCTACACTTCTGGA-SH
	N1	GGGGAGTATTGCGGAGTTGTGGATATTTAGTGGAGATGTAG
	N2	TGGACGGGGAGTATTGTAAAGTAGTAGGTCGTAGGGTATTGAAT
	N3	TAGAATACAGAAGTGCGTTTGTGGATATTTAGTGGAGATG
	N4	TAGAATACAGAAGTGCGTTTAAAGTAGTAGGTCGTAGGGTATTGAAT
	N5	TAAAGTAGTAGGTCGTAGGGTATTGAATGTGGAGATGTAG
	N6	TGGACGGGGAGTATTGTAAAGTAGTAGGTCGTAGGGTATTGAATGTGGAGATG
	i-rl1	CTCCCTCCGCAATACTCCCCGTCCATGTA-SH
	i-rl2	CGACCTACTACTTTATGTC-SH
	InA	GTGGAGATGTAGGAAAG
	InB	ATTCAATACCCTACGACCTACTACTTTA
	InN	CATGAGGGGCGGGGACGCCGCCTGTCT
8, 9, S11	i/o-r1	NH2-CTTTCCTACATCTCCAC-Bio
	i/o-r2	NH2-CTTTCCTACATCTCCACATTCAATACCCTACG-Bio
	N1	GGGGAGTATTGCGGAGTTGTGGATATTTAGTGGAGATGTAG
	N2	TGGACGGGGAGTATTGTAAAGTAGTAGGTCGTAGGGTATTGAATGTGGAGATG
	InA	GTGGAGATGTAGGAAAG
	InB	ATTCAATACCCTACGACCTACTACTTTA
	InN	CATGAGGGGCGGGGACGCCGCCTGTCT

Table S2. Characteristics of microspheres tested as core particles for DTs construction

Brand	Type	Size, µm	Functional Groups	Conjugation Conditions
Dynabeads Tosylactivated (Thermo Fisher Scientific Inc., USA)	MyOne	1.0	Tosyl	Direct immobilization in borate buffer, pH 9.4
	M-280	2.8		
Estapor (Merck Millipore Inc., USA)	Incapsulated Fe_3O_4	1.0	Carboxylic	Carbodiimide pre-activation
	Non-incapsulated Fe_3O_4	0.3		
		2.0		
Spherotech (Spherotech Inc., USA)	Smooth	3.4		
	Non-smooth	3.7		

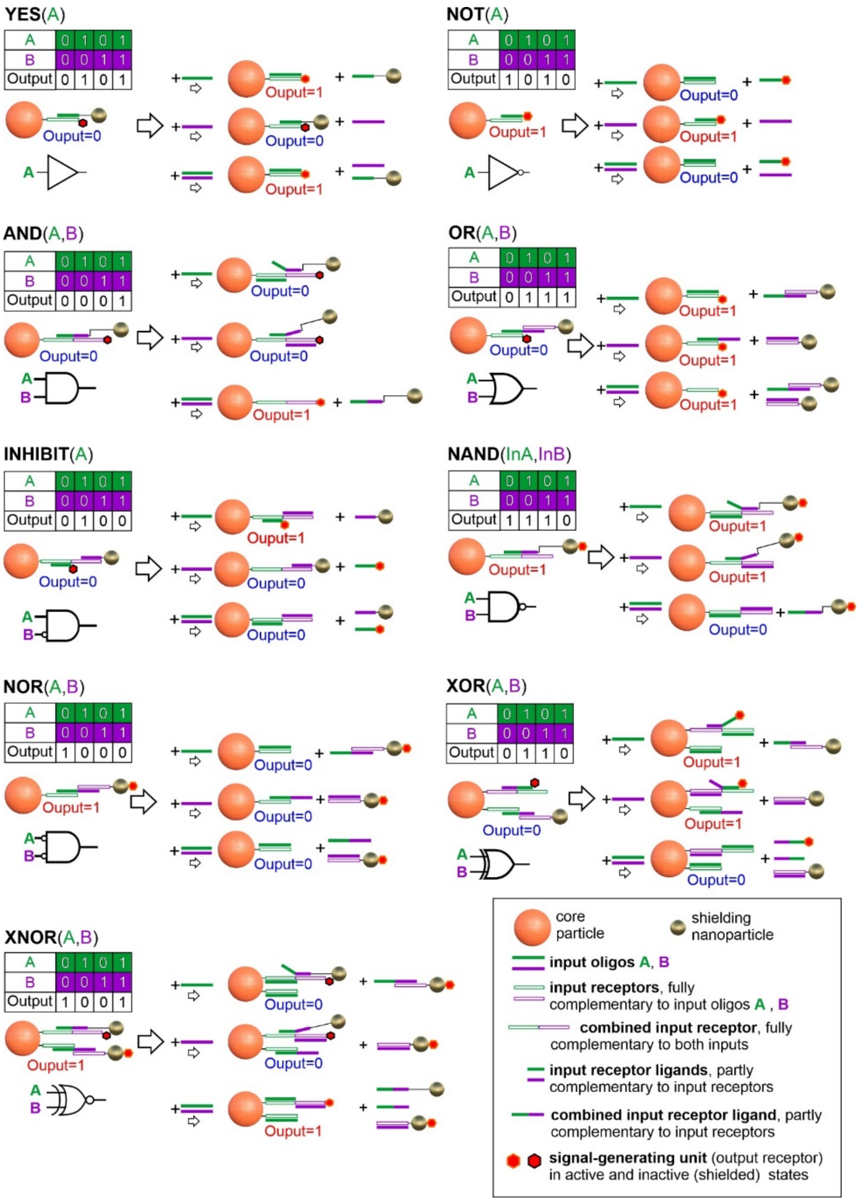

Figure S1. The scheme of the composite logic gate operating using toehold-mediated strand displacement on the DT surface.

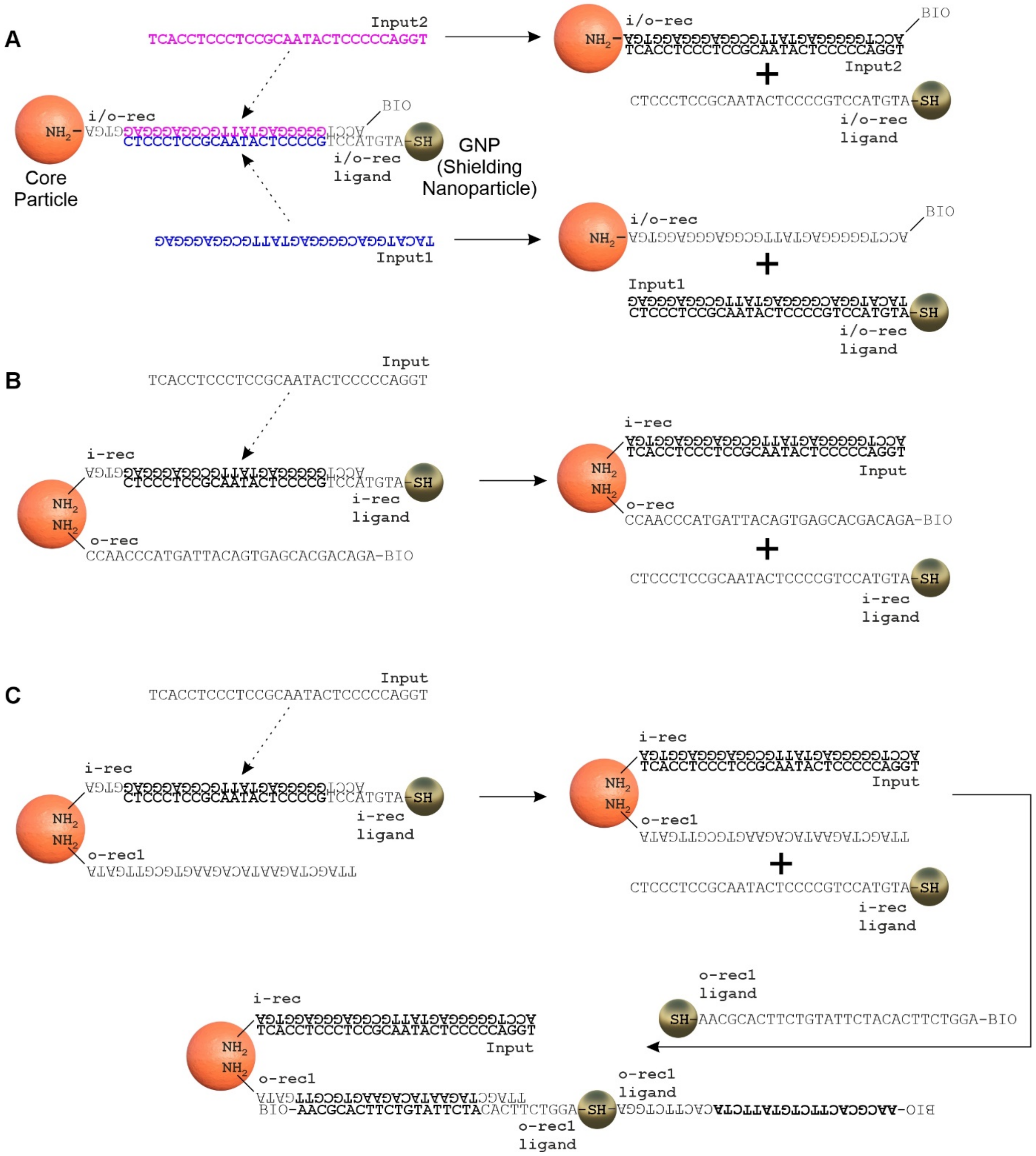

Figure S2. Variants of constructing a logic YES gate based on DTs, implemented in this study (see **Figure 3** for details).

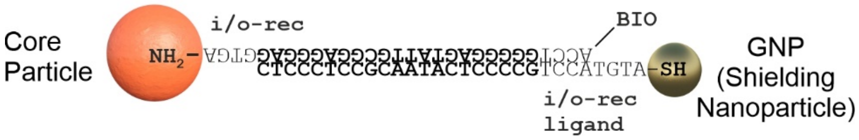

Figure S3. Experimental schemes utilized to evaluate the efficiency of core and shielding particle binding, as well as the indirect assessment of the degree of shielding using scanning electron microscopy. For further details, please refer to **Figure 4A**.

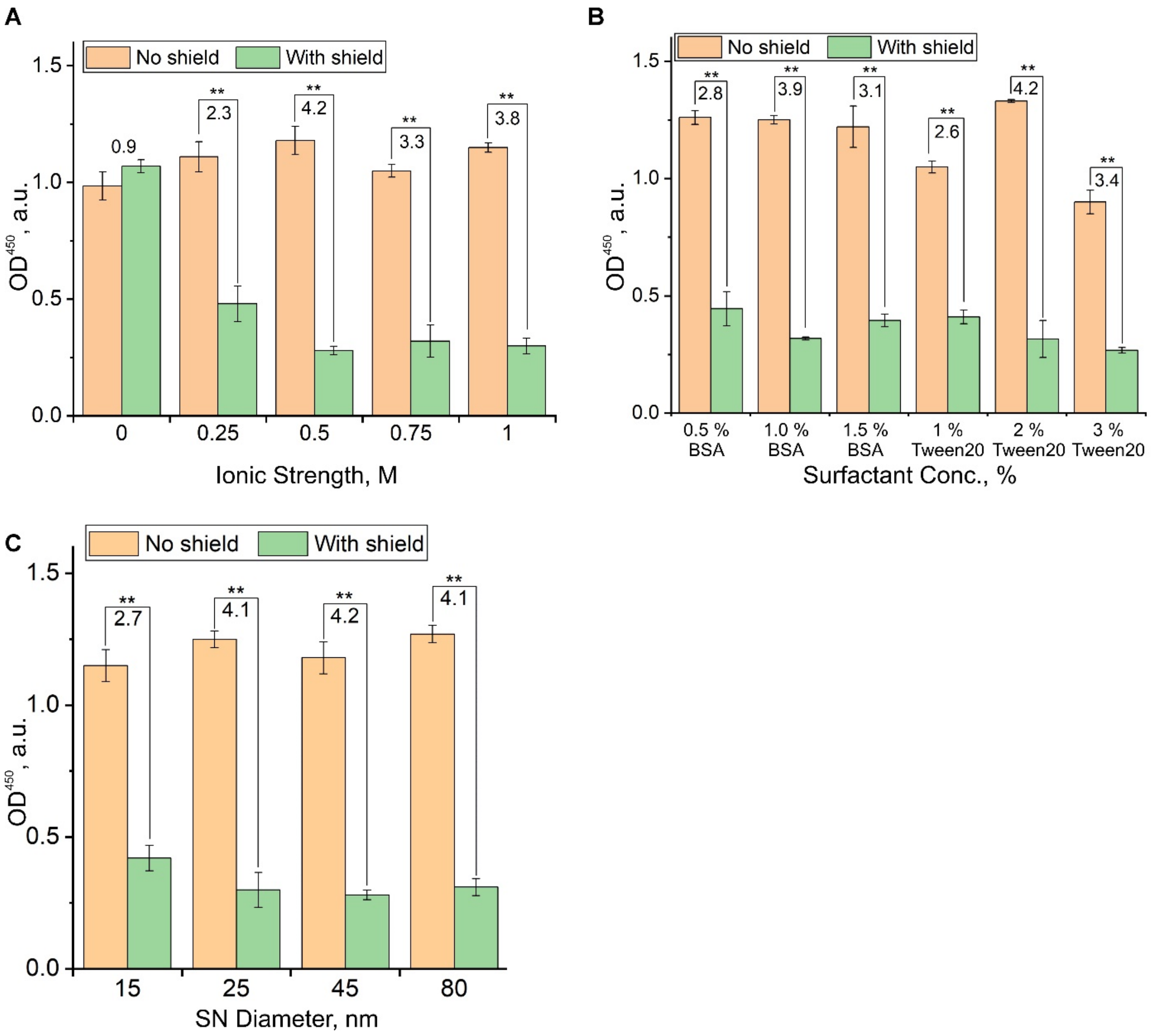

Figure S4. Selective results on optimization of DT assembly conditions for implementing the YES gate according to the scheme in **Figure 3A**. The figure displays the DT optical output as a function of: (A) ionic strength (NaCl concentration); (B) the composition of the working buffer; (C) the size of the shielding particles at a fixed concentration (0.15 M). The significance level of the difference ($p < 0.05$ and 0.01) between shielded and unshielded states of DTs are indicated by one and two asterisks, respectively. The numbers provided indicate the value of the SGS signal ratio between the initial and shielded states.

S1. Evaluation of the performance of input and output ligands during their co-immobilization on the core particles (CPs).

To evaluate the concurrent presence and specificity of immobilized input and output oligonucleotides on the core particles (CPs), we employed Förster quenching to screen the optically active end-marks of output receptors with gold nanoparticles, taking advantage of the known interaction between fluorophores and the gold nanoparticle (**Figure S5A**).

To confirm the presence of immobilized input and output oligonucleotides (i-rec and o-rec, respectively) on the surface of CPs, we utilized a 5'-ROX dye (6-carboxy-X-rhodamine) to modify the output oligonucleotide, o-rec(ROX). The fluorescence of the o-rec(ROX) was assumed to indicate the presence of the output receptor on the initial CP-ROX particles. Fluorescence was measured using fluorescence microscopy (Axio Observer A1, Carl Zeiss, Germany) and validated using LumoTrace FLUO bioimaging system (Abisense, Russia). The quenching of fluorescence after incubation with the Sn-spec conjugate indicated the close proximity of the input ligand (i-rec) to the output ligand (o-rec(ROX)), i.e., their co-localization on the surface of the CP at a distance less than the Förster radius.

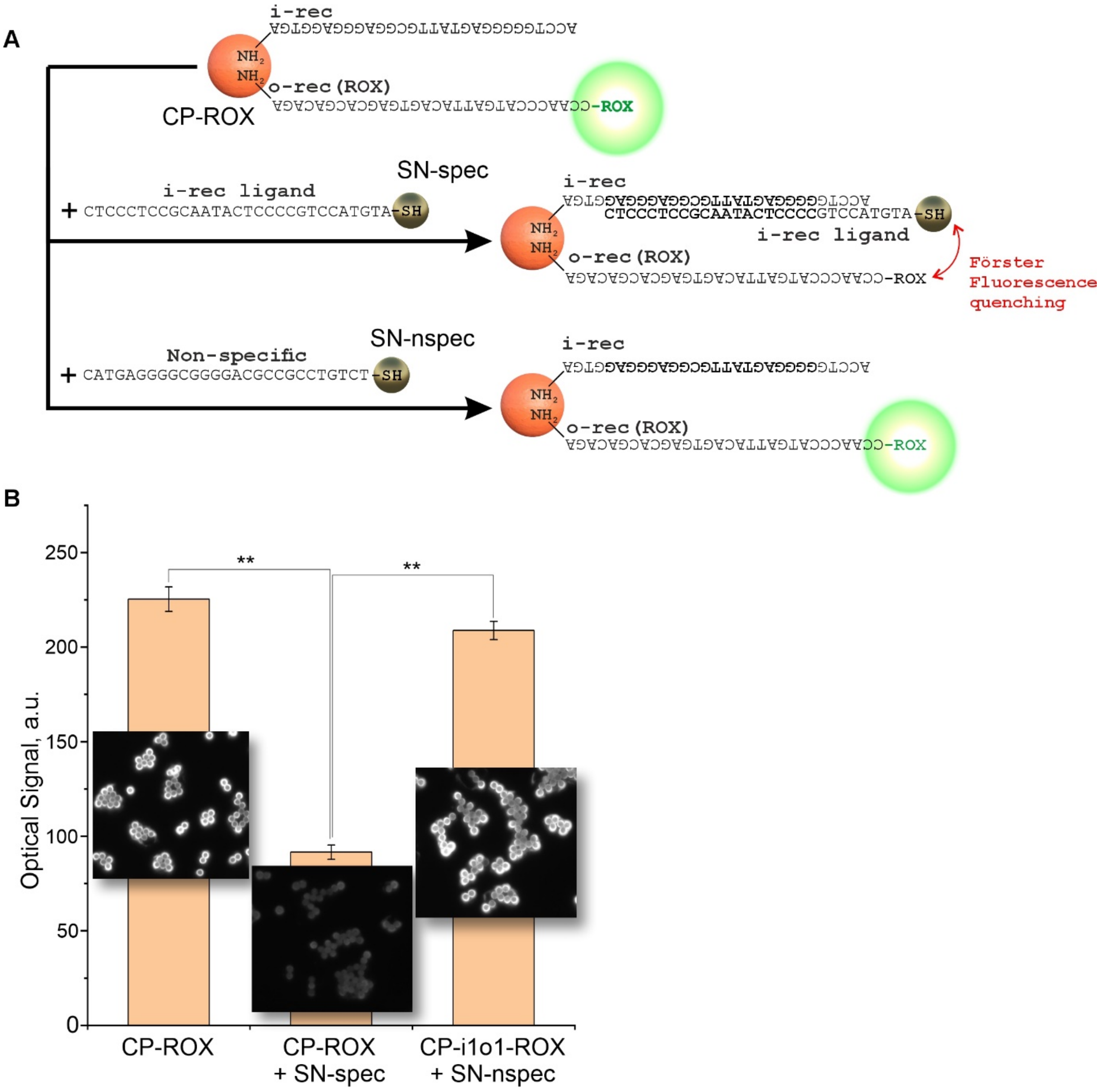

Figure S5. Verification of simultaneous immobilization and accessibility of input (i-rec) and output (o-rec) oligonucleotides on the surface of the Core Particle (CP). (A) Scheme of the

experiment using the effect of fluorescence quenching by gold nanoparticles when they are closely located with a fluorophore (ROX dye) at the end of the output ligand. (B) Dependence of the fluorescence intensity of CP with co-immobilized i-rec and o-rec(ROX) oligonucleotides (CP-ROX) on the presence of shielding gold nanoparticles (SN) with immobilized i-rec ligand (specific to i-rec, SN-spec) or nonspecific oligonucleotides (SN-nspec). The Y-axis on the graph shows the result of processing optical microphotographs (see insets) using ImageJ software.

The data (**Figure S5B**) revealed that the initial CP-ROX particles exhibited strong fluorescence, which was only quenched in the presence of gold nanoparticles with oligonucleotides complementary to the input receptors. This observation confirms the co-localization of the input and output oligonucleotides on the surface of the CP, thus demonstrating the feasibility of constructing a Signal Generation System (SGS) based on separately immobilized ligands.

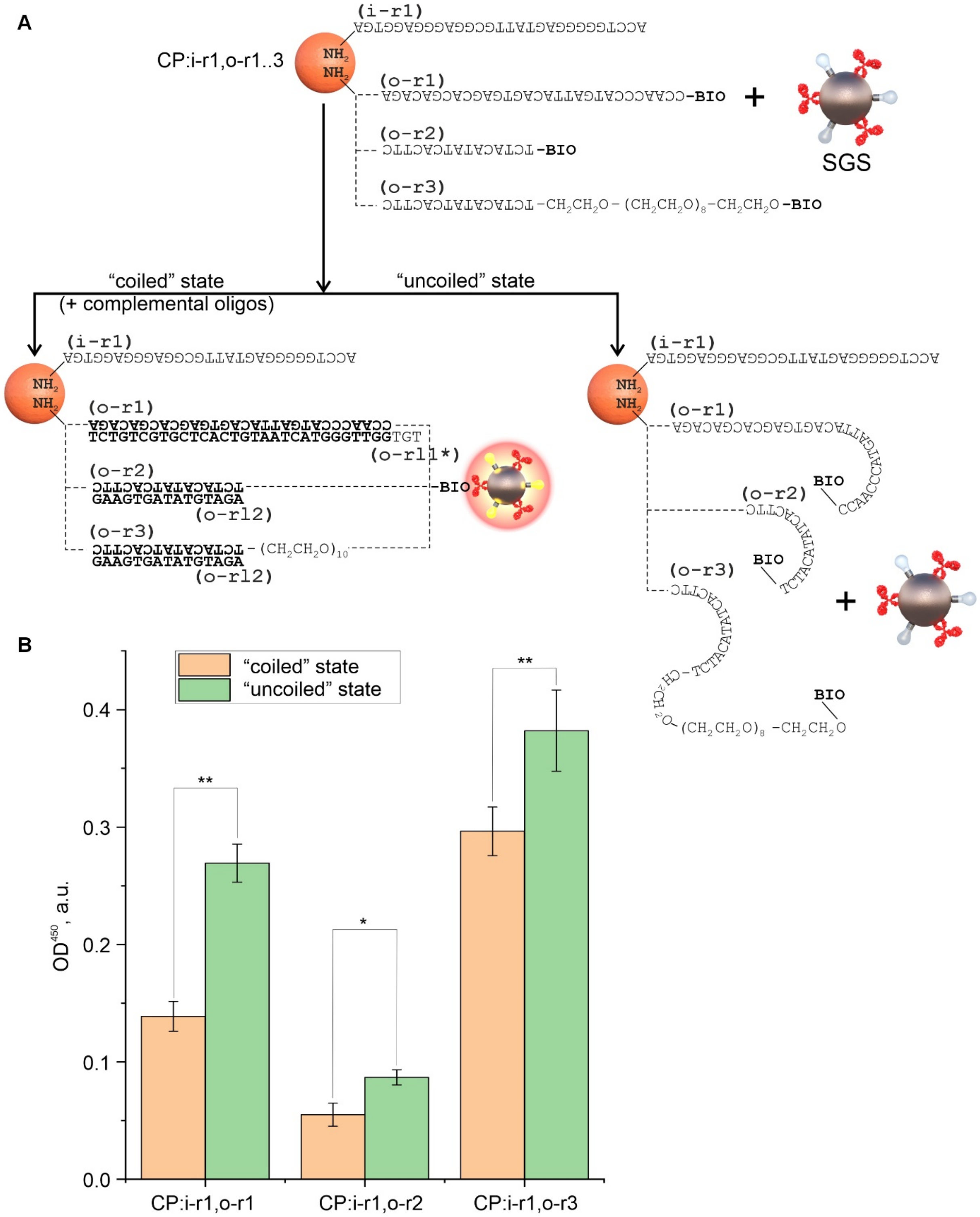

Figure S6. The impact of output oligonucleotide length on DT signal during co-immobilization of input and output oligonucleotides. (A) Experimental design. Signal generation system (SGS) based on ferrihydride nanoparticles-HRP-Str conjugate and hydrogen peroxide/TMB; (B) Optical signal of DT/SGS interaction depending on with varying output oligonucleotide length. Results are presented for three output receptor oligonucleotides: o-r1 (30 nt), o-r2 (15 nt), and o-r3 (15 nt + 10 PEG unit spacer) in both the initial single-stranded ("coiled") and hybridized double-stranded ("uncoiled") states following

hybridization with corresponding complementary oligonucleotides (o-rl1, o-rl2, o-rl3). One and two asterisks indicate the significance level of the difference ($p < 0.05$ and 0.01).

The data obtained show that, on the whole, the magnitude of the signal is proportional to the length of the spacer chain. At the same time, for this system, we observed the previously discovered effect of "masking" the terminal fragments of single-stranded DNA immobilized on the surface of nanoparticles that were hybridized with complementary sequences. Apparently, this effect is associated with a noticeable stiffening of the oligonucleotide chain after hybridization. As a result, an increase in the accessibility of the output terminal receptors, sterically shielded initially, is achieved. Obviously, this effect must be taken into account when designing molecular gates, since under certain conditions it can lead to distortions of the resulting signal (for example, due to conformational fluctuations of the molecules of the output oligonucleotides under the influence of environmental conditions, molecular environment, etc.). When such an effect is manifested, it is possible to change the scheme for generating the output signal, using the SGS variant described above with the combination of input and output ligands in one and the same oligonucleotides (**Figure 3A**), or the variant with signal amplification (**Figure 3C**), which showed much more reproducible results and was, in particular, further used to build more complex gates (see **Main Manuscript, Part 3**).

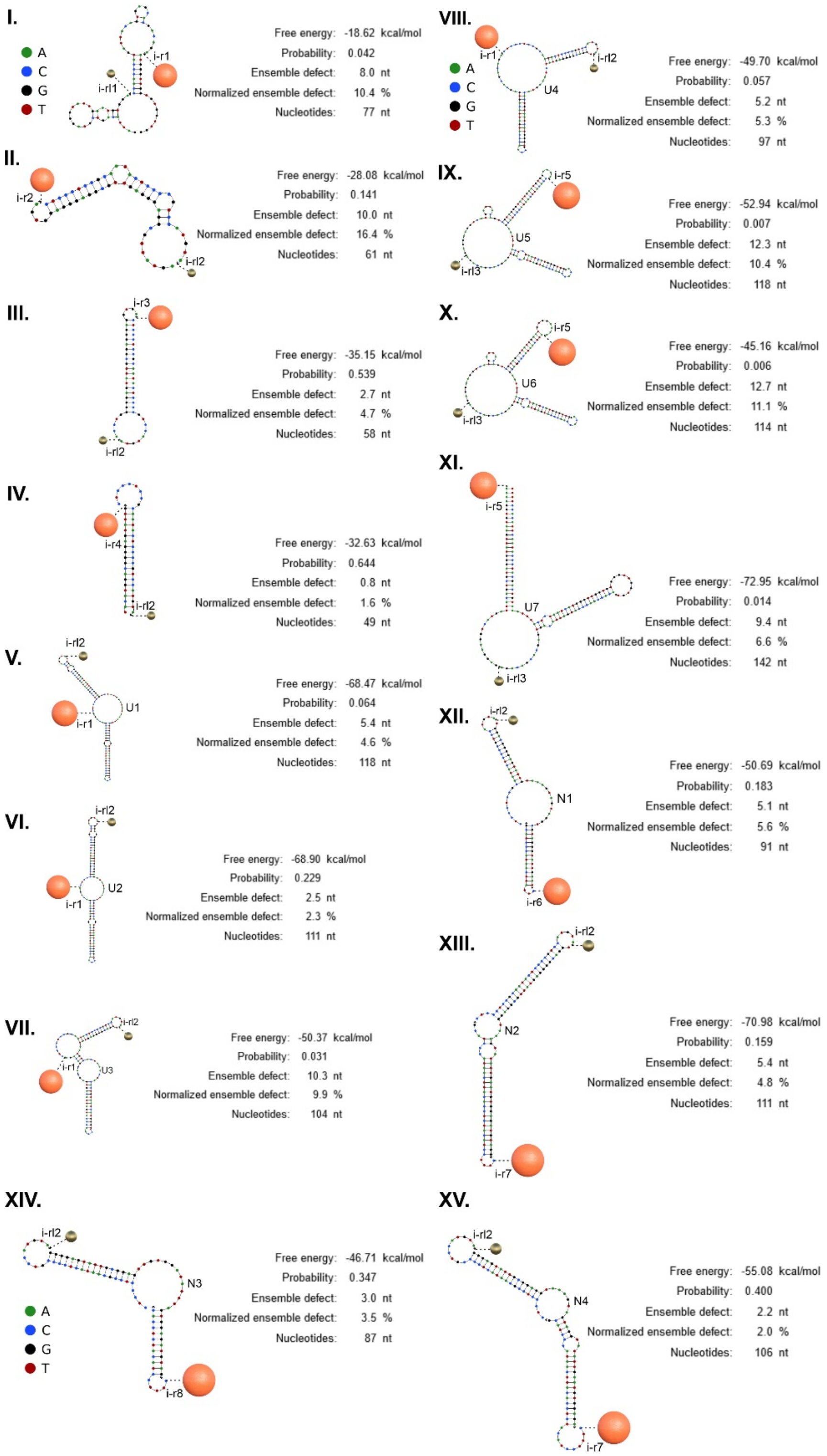

Figure S7. Schematic representation of the fifteen tested variants (I to XV) for constructing DTs to implement a YES gate using a two-component SGS and randomly generated nucleotide sequences. Data obtained from Nupack software (http://www.nupack.org/).

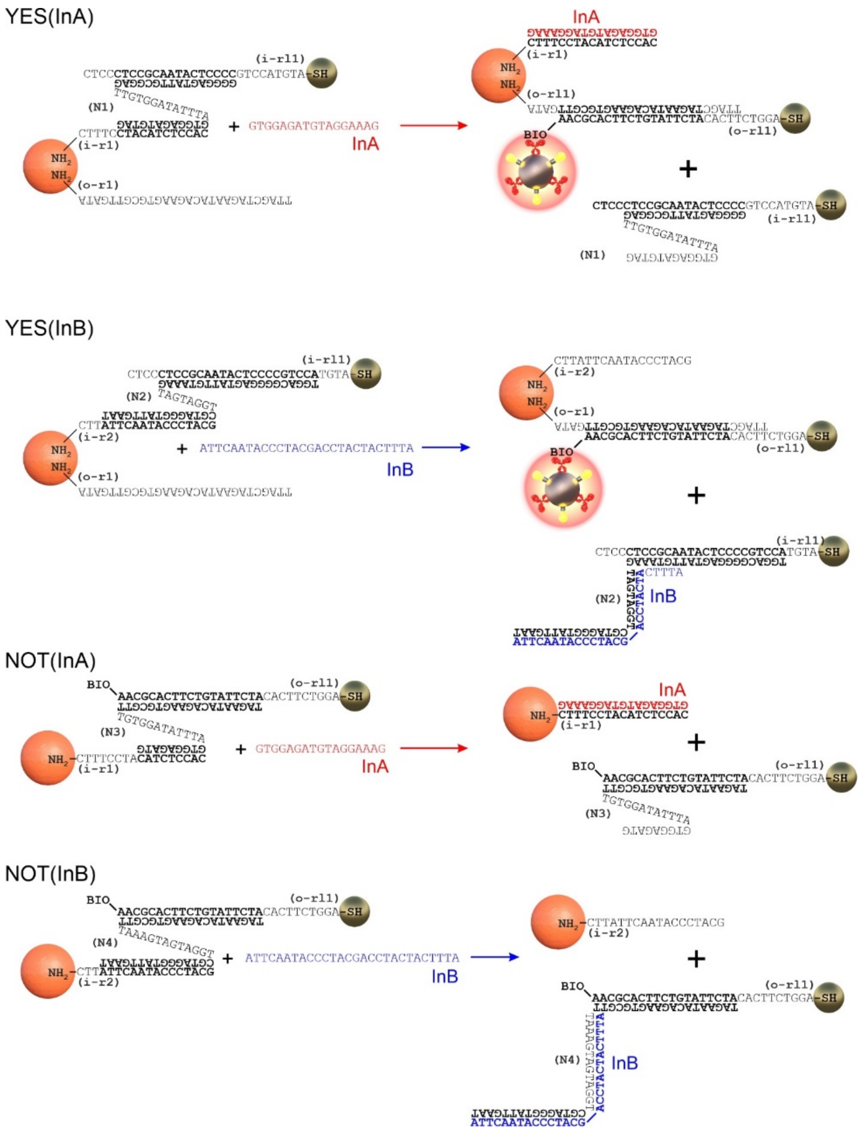

Figure S8. Implementation schemes for YES and NOT gates (see **Figure 6** for details).

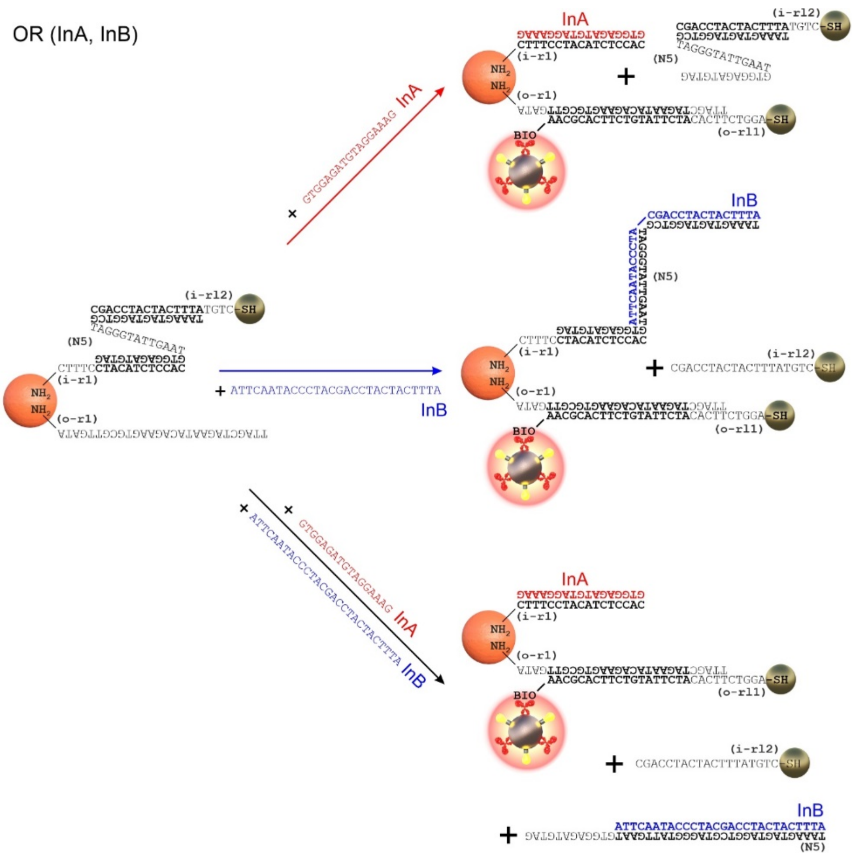

Figure S9. Implementation schemes for OR gate (see **Figure 7A** for details).

AND (InA, InB)

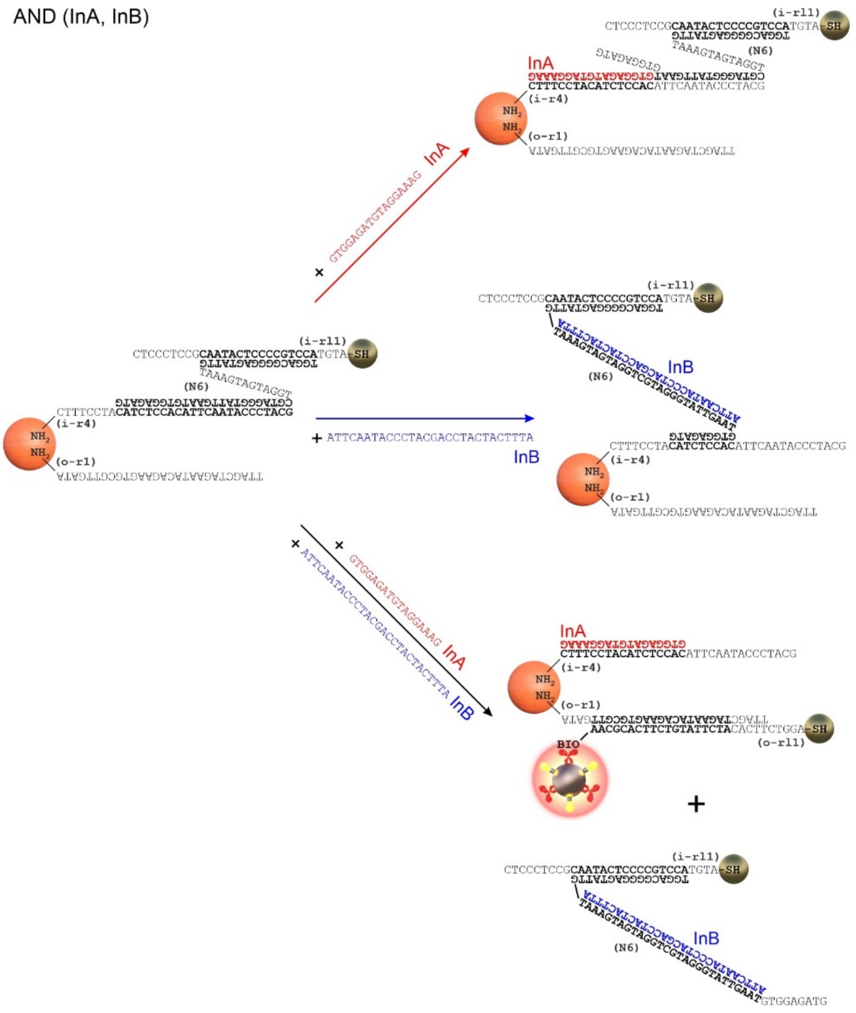

Figure S10. Implementation schemes for AND gate (see **Figure 7B** for details).

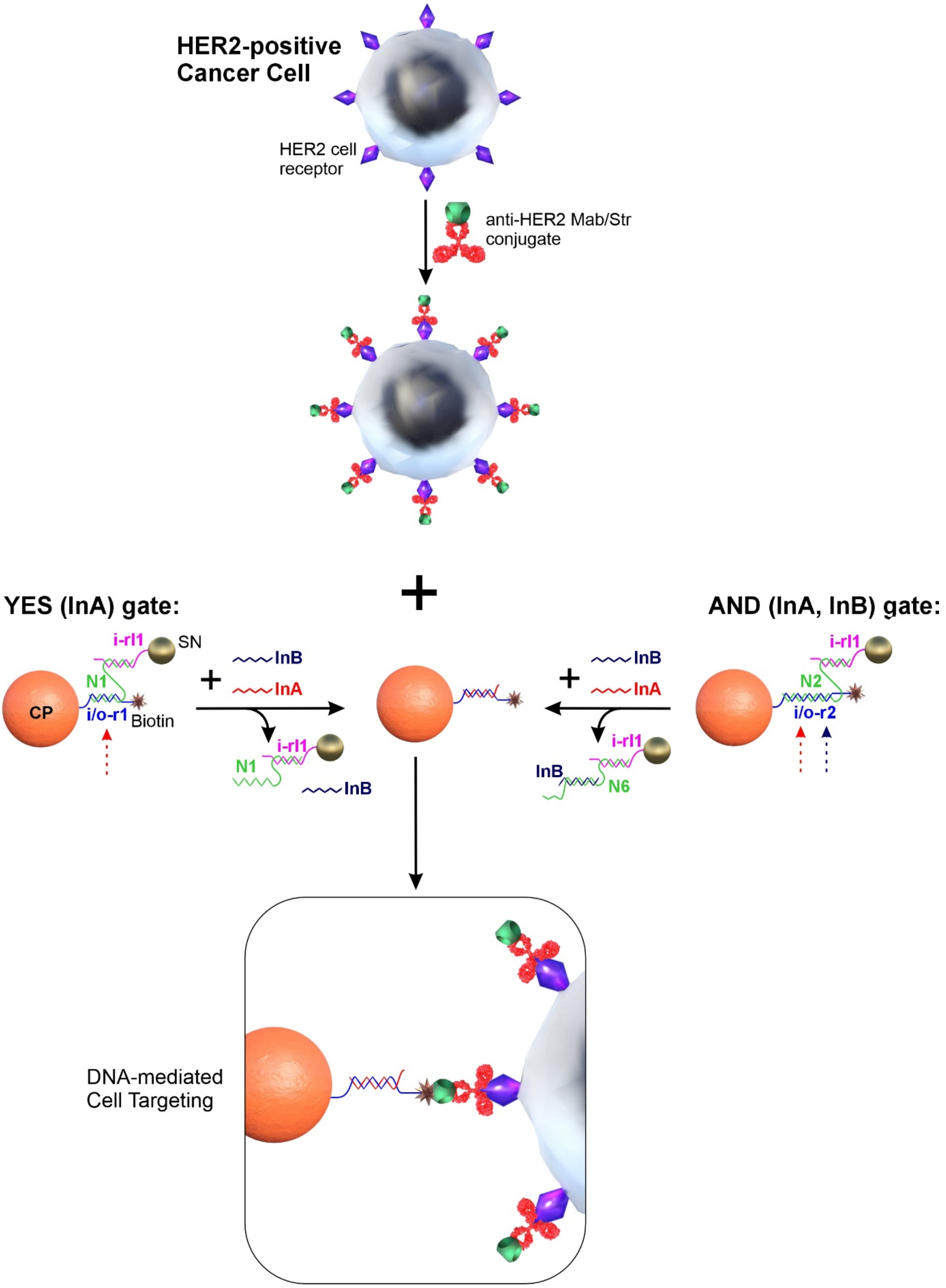

Figure S11. Scheme for implementing DNA-mediated targeting of HER2-positive cells as a result of YES and AND gating. The sequences of the oligonucleotides used are shown in **Table S1**.
